# Genomic Convergence among ERRα, PROX1, and BMAL1 in the Control of Metabolic Clock Outputs

**DOI:** 10.1371/journal.pgen.1002143

**Published:** 2011-06-23

**Authors:** Catherine R. Dufour, Marie-Pier Levasseur, Nguyen Hoai Huong Pham, Lillian J. Eichner, Brian J. Wilson, Alexis Charest-Marcotte, David Duguay, Jean-François Poirier-Héon, Nicolas Cermakian, Vincent Giguère

**Affiliations:** 1 Goodman Cancer Research Centre, Montréal, Canada; 2 Department of Biochemistry, McGill University, Montréal, Canada; 3 Laboratory of Molecular Chronobiology, Douglas Mental Health University Institute, Montréal, Canada; 4 Department of Psychiatry, McGill University, Montréal, Canada; 5 Department of Neurology and Neurosurgery, McGill University, Montréal, Canada; 6 Department of Medicine, McGill University, Montréal, Canada; 7 Department of Oncology, McGill University, Montréal, Canada; University of Texas Southwestern Medical Center, United States of America

## Abstract

Metabolic homeostasis and circadian rhythms are closely intertwined biological processes. Nuclear receptors, as sensors of hormonal and nutrient status, are actively implicated in maintaining this physiological relationship. Although the orphan nuclear receptor estrogen-related receptor α (ERRα, NR3B1) plays a central role in the control of energy metabolism and its expression is known to be cyclic in the liver, its role in temporal control of metabolic networks is unknown. Here we report that ERRα directly regulates all major components of the molecular clock. ERRα-null mice also display deregulated locomotor activity rhythms and circadian period lengths under free-running conditions, as well as altered circulating diurnal bile acid and lipid profiles. In addition, the ERRα-null mice exhibit time-dependent hypoglycemia and hypoinsulinemia, suggesting a role for ERRα in modulating insulin sensitivity and glucose handling during the 24-hour light/dark cycle. We also provide evidence that the newly identified ERRα corepressor PROX1 is implicated in rhythmic control of metabolic outputs. To help uncover the molecular basis of these phenotypes, we performed genome-wide location analyses of binding events by ERRα, PROX1, and BMAL1, an integral component of the molecular clock. These studies revealed the existence of transcriptional regulatory loops among ERRα, PROX1, and BMAL1, as well as extensive overlaps in their target genes, implicating these three factors in the control of clock and metabolic gene networks in the liver. Genomic convergence of ERRα, PROX1, and BMAL1 transcriptional activity thus identified a novel node in the molecular circuitry controlling the daily timing of metabolic processes.

## Author Summary

The molecular basis for coordinated control of circadian rhythms and metabolism is not well understood. Although integral components of the molecular clock such as the transcription factor BMAL1 can directly regulate some metabolic genes, the output from the circadian oscillator is believed to be in large part mediated through the action of transcription factors whose patterns of expression are rhythmic in metabolic tissues. The estrogen-related receptor α (ERRα, NR3B1) and its corepressor PROX1, two major metabolic regulators, could be well suited for this function. Indeed, we show that proper maintenance of daily glucose, insulin, bile acid, lipid, and locomotor rhythms in mice are dependent on the presence of ERRα. Ablation of PROX1 in synchronized HepG2 cells revealed the importance of PROX1 in regulating the rhythmic expression of clock and metabolic genes. Using genome-wide analysis of promoter occupancy and gene expression analyses, we identify ERRα and PROX1 as novel transcriptional regulators of the molecular clock and show that the direct participation of BMAL1 in the clock output pathway related to metabolic control is highly extensive. ERRα and BMAL1 thus serve as key bidirectional regulators connecting the peripheral clock and cellular energy metabolism.

## Introduction

In most living organisms, metabolic and behavioral processes are orchestrated in a timely fashion approximating a 24 hr daily cycle. In mammals, light/dark (LD) cycles regulate the diurnal activity of the master pacemaker within the suprachiasmatic nuclei (SCN) and in turn synchronize autonomous molecular clocks in peripheral tissues [1]–[3]. A small network of core clock genes coordinate the initiation and regulation of the circadian expression of genes and are interconnected by positive and negative transcriptional and translational feedback loops [4]. The primary loop is comprised of the positive transcriptional regulators BMAL1 and CLOCK and the transcriptional repressors PERIOD (PER) and CRYPTOCHROME (CRY) [5]–[7]. Upon heterodimerization, BMAL1 and CLOCK work together to activate the cyclic expression of core clock genes and mediators of the molecular clock called clock-controlled genes (CCGs). PER and CRY proteins function to repress BMAL1/CLOCK transcriptional activity to ensure the continous daily rhythmic expression of genes. Integrity of the mammalian clock is vital as dysfunction in the timed oscillation of genes has been associated with various diseases including obesity and cancer [8], [9].

The peripheral clock in metabolic tissues such as liver is reset by physiological cues such as food availability [10], [11] and CCG networks are responsible for the circadian timing of metabolic processes including glucose homeostasis, fatty acid oxidation and cholesterol synthesis and degradation [9], [12]–[14]. Although integral components of the molecular clock can directly regulate some metabolic genes, the output from the circadian oscillator is believed to be in large part mediated through the action of transcription factors whose patterns of expression are rhythmic in metabolic tissues [15]. In this regard, members of the nuclear receptor superfamily are well suited for this function. Nuclear receptors can translate nutrient and hormone signals into specific expression signatures of metabolic genes and several members of the family are expressed in a rhythmic fashion in metabolic tissues [16], [17]. Indeed, evidence of functional cross-talk between various nuclear receptors and core clock genes is rapidly accumulating. First, nuclear receptors such as REV-ERBα and REV-ERBβ (NR1D1 and NR1D2) and RORα, β and γ (NR1F1, NR1F2 and NR1F3) are directly linked to BMAL1 and CLOCK via interconnecting positive and negative transcriptional feedback loops [18]–[20]. Second, nuclear receptors implicated in metabolic control such as PPARα (NR1C1) and TRα (NR1A1) have been shown to act as indirect mediators of BMAL1 and CLOCK to carry out specific metabolic outputs in a circadian manner [21], [22]. Third, PER2 has recently been shown to propagate clock information to metabolic genes by directly interacting with and acting as a coregulator of nuclear receptor-mediated transcription [23].

The orphan nuclear receptor estrogen related receptor α (ERRα, NR3B1) plays a critical role in the control of cellular energy metabolism [24], [25]. In addition, ERRα transcripts display a diurnal rhythm in several tissues including liver, kidney, uterus and bone [17], [26], [27]. The transcriptional activity of ERRα is highly dependent on interactions with coregulatory proteins, most notably members of the family of PGC-1 coactivator proteins [28]–[32]. Interestingly, PGC-1α and PGC-1β were shown to be rhythmically expressed in liver and skeletal muscle and PGC-1α was shown to enhance the expression of molecular clock genes [33]. The rhythmic expression of clock and metabolic genes were altered in PGC-1α-null mice consequently resulting in abnormal circadian physiological rhythms including locomotor activity, body temperature and metabolic rate. Conversely, we have recently shown that the metabolic function of the ERRα/PGC-1α complex can be antagonized in liver cells by the transcriptional regulator Prospero-related homeobox 1 (PROX1) [34]. PROX1 directly interacts with both ERRα and PGC-1α, represses the transcriptional activity of the ERRα/PGC-1α complex on metabolic gene promoters and opposes the effects of ERRα on the respiratory capacity of liver cells in culture. In support of these observations, functional genomic analyses using a ChIP-on-chip approach in mouse liver revealed that ERRα and PROX1 share approximately 50% of their target genes. These common targets include a broad range of metabolic genes involved in carbohydrate and fatty acid metabolism, tricarboxylic acid cycle (TCA) cycle, electron transport and oxidative phosphorylation (OXPHOS) [34]. PROX1 has also been recently identified as a genetic locus implicated in fasting glucose homeostasis in human subjects [35]. Whether ERRα and PROX1 participate in the rhythmic control of metabolism and/or have functional interaction with integral components of the molecular clock is currently unknown.

In this report, we first show that proper maintenance of diurnal glucose, insulin, bile acid, cholesterol, non-esterified fatty acid (NEFA) and triglyceride levels as well as locomotor rhythms in mice is dependent on the presence of ERRα. Analyses of ChIP-on-chip and gene expression datasets as well as functional studies further demonstrate that ERRα PROX1 and BMAL1 are involved in both transcriptional regulatory loops and rhythmic control of components of all major metabolic pathways in the liver. In particular, we show that BMAL1 directly targets a significantly large number of genes linked to diverse biological processes, including cellular metabolism. Our results thus demonstrate that BMAL1 plays a more comprehensive role in the circadian output pathways than previously anticipated and uncover a novel node in the intricate transcriptional network necessary to sustain proper metabolic and circadian rhythms.

## Results

### ERRα regulates diurnal glucose homeostasis

**Figure 1:**
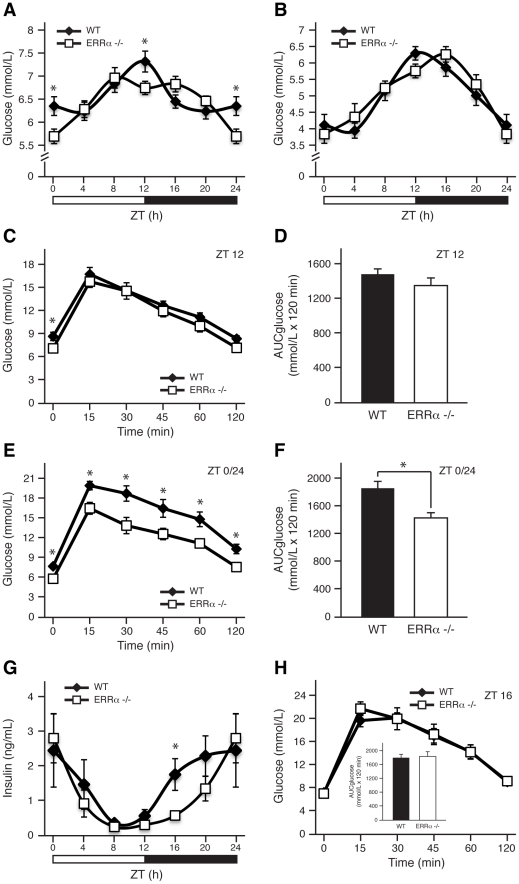
ERRα is involved in diurnal glucose homeostasis. Blood glucose measurements taken from fed *ad libitum* (A) and fasted (B) male WT and ERRα-null mice (n = 15 and n = 8 for A and B respectively) at 4 hr intervals over a 24 hr period from ZT 4 to ZT 24. Error bars represent ± SEM. Student's t test, *p<0.05. ZT 0 values are a duplicate of ZT 24 shown for clarity. (C) Blood glucose during a glucose tolerance test in WT and ERRα-null mice (n = 8) at ZT 12 following a 6 hr fast and intraperitoneal glucose administration of 2 mg/g body weight. Error bars represent ± SEM. Student's t test, *p<0.05. (D) Area under the glucose curve at ZT 12. Error bars represent ± SEM. (E) Blood glucose during a glucose tolerance test in WT and ERRα-null mice (n = 6) at ZT 0/24 following a 6 hr fast and intraperitoneal glucose administration of 2 mg/g body weight. Error bars represent ± SEM. Student's t test, *p<0.05. (F) Area under the glucose curve at ZT 0/24. Error bars represent ± SEM. Student's t test, *p<0.05. (G) Diurnal serum insulin levels in fed *ad libitum* WT and ERRα-null mice. Error bars represent ± SEM. Student's t test, *p<0.05. ZT 0 values are a duplicate of ZT 24 shown for clarity. (H) Blood glucose during a glucose tolerance test in WT and ERRα-null mice (n = 6) at ZT 16 following a 6 hr fast and intraperitoneal glucose administration of 2 mg/g body weight. Error bars represent ± SEM. Inset: area under the glucose curve at ZT 16. Error bars represent ± SEM.

We have recently observed that mice lacking ERRα have impaired diurnal blood pressure levels that are associated with changes in the expression of Na^+^ and K^+^ transporters in the kidneys [27]. These results indicate that ERRα could be involved in orchestrating other physiological and behavioral processes in a timely fashion. Given the importance of ERRα in the regulation of genes involved in glycolysis and gluconeogenesis [34], we first sought to compare blood glucose levels in wild-type (WT) and ERRα-null mice fed *ad libitum* across the day. As expected [8], WT mice display noticeable diurnal variation of glucose levels in circulating blood (Figure 1A). However, mice lacking ERRα had significantly lower glucose levels at Zeitgeber times (ZT) 12 and ZT 0/24, coinciding with the start and end of the dark cycle, respectively (Figure 1A). The observed hypoglycemia may be due to altered glucose uptake by muscle and fat and/or glucose output from the liver. Food availability appears to be a major determinant in the observed differences in circulating glucose levels in ERRα-null mice as the time-dependent hypoglycemia was lost under fasting conditions (Figure 1B). Subsequently, we performed glucose tolerance tests at ZT 12 and ZT 0/24 to investigate whether differences in insulin secretion and response could account for the deregulated glucose homeostasis observed in fed ERRα-null mice. At ZT 12 following a 6 hr fast, basal glucose measurements were significantly lower in ERRα-null mice but no difference in glucose levels were seen post-glucose injection (Figure 1C). In Figure 1D, the data is illustrated as the total area under the curve (AUC_glucose_) calculated using the trapezoidal rule. In contrast, glucose tolerance tests at ZT 0/24 revealed that ERRα-null mice have improved glucose handling demonstrated by significantly decreased basal blood glucose levels after a 6 hr fast and at each subsequent time-point post-glucose administration (Figure 1E and 1F, p = 0.0046). Our data suggest that the hypoglycemia observed in ERRα-null mice under *ad libitum* feeding may in part be due to enhanced insulin secretion and/or response. We thus measured circulating serum insulin levels and determined however that fed ERRα-null mice have significantly reduced insulin levels at ZT 16 (Figure 1G). The delay in insulin secretion during the dark cycle observed in ERRα-null mice reflects the trend towards latency in glucose uptake seen under fed *ad libitum* conditions (Figure 1A and 1G). Glucose tolerance tests at ZT 16 were performed to determine whether the lower circulating insulin levels in ERRα-null mice at this time would result in impaired glucose tolerance. As shown in Figure 1H, loss of ERRα expression in mice had no impact on glucose tolerance at ZT 16.

**Figure 2:**
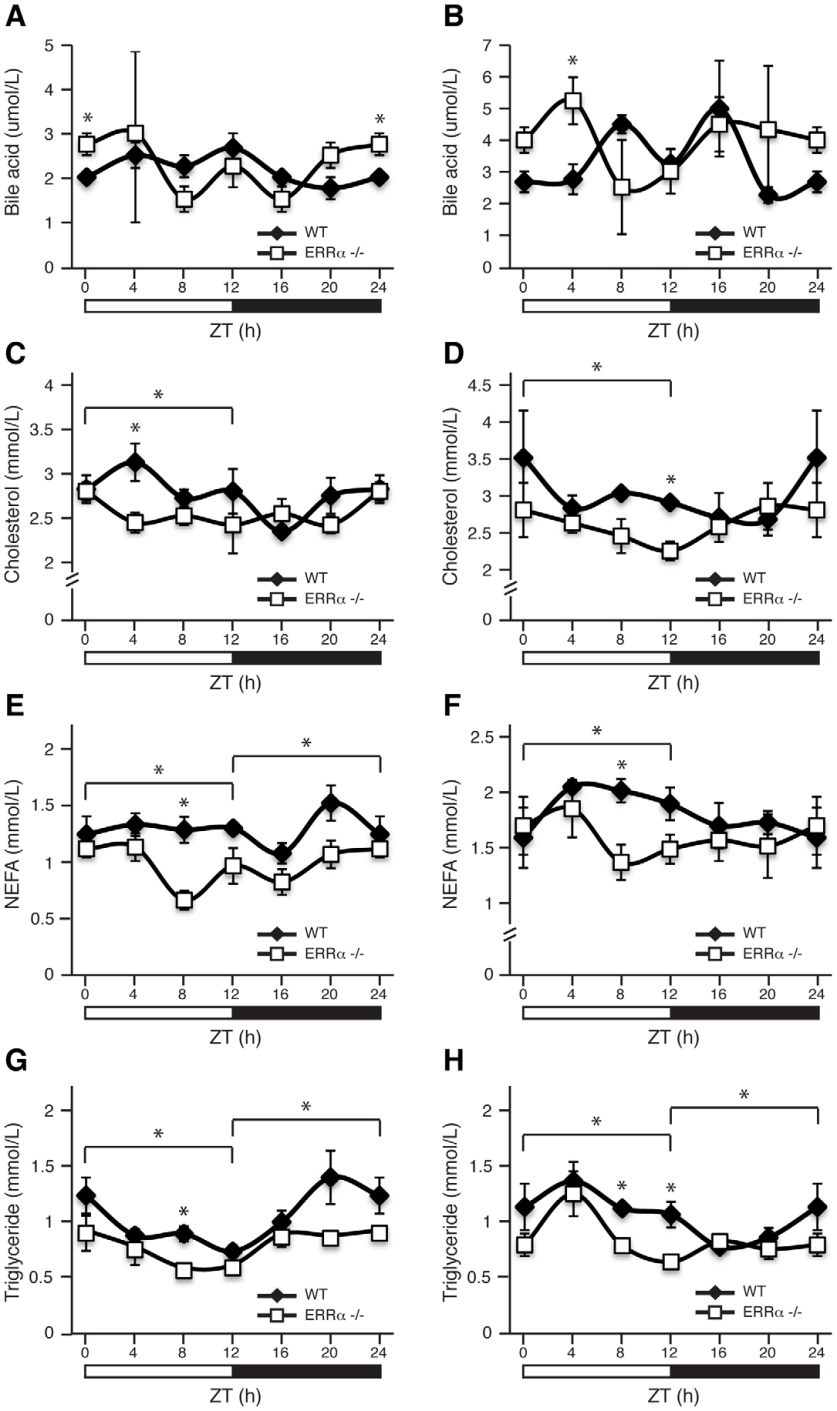
Diurnal variation of serum bile acid, cholesterol, NEFA, and triglyceride levels in WT and ERRα-null mice. Circulating metabolic parameters were measured in fed *ad libitum* and fasted WT and ERRα-null mice (n = 4) at 4 hrs intervals over a 24 hr period from ZT 4 to ZT 24. Error bars represent ± SEM. Student's t test, *p<0.05 at individual time points or across a 12 hr light or dark cycle. ZT 0 values are a duplicate of ZT 24 shown for clarity. (A–B) Fed and fasted bile acid, (C–D) fed and fasted total cholesterol, (E–F) fed and fasted NEFA and (G–H) fed and fasted triglyceride levels are shown.

Next, we investigated whether ERRα expression is important in maintaining the diurnal levels of other circulating metabolites. Bile acid, total cholesterol, NEFA and triglycerides were measured in both fed and fasted mice. ERRα-null mice were found to have significantly greater serum bile acids at ZT 0/24 and ZT 4 under fed and fasted conditions, respectively (Figure 2A and 2B). On the other hand, ERRα-null mice have less circulating cholesterol, NEFA and triglycerides determined at least at one time point during a 24 hr cycle regardless of food availability (Figure 2C–2H). Differences in these circulating metabolite levels in both fed and fasted ERRα-null mice are more apparent when measurements are compiled throughout a complete 12 hr light or dark cycle (Figure 2C–2H).

**Figure 3:**
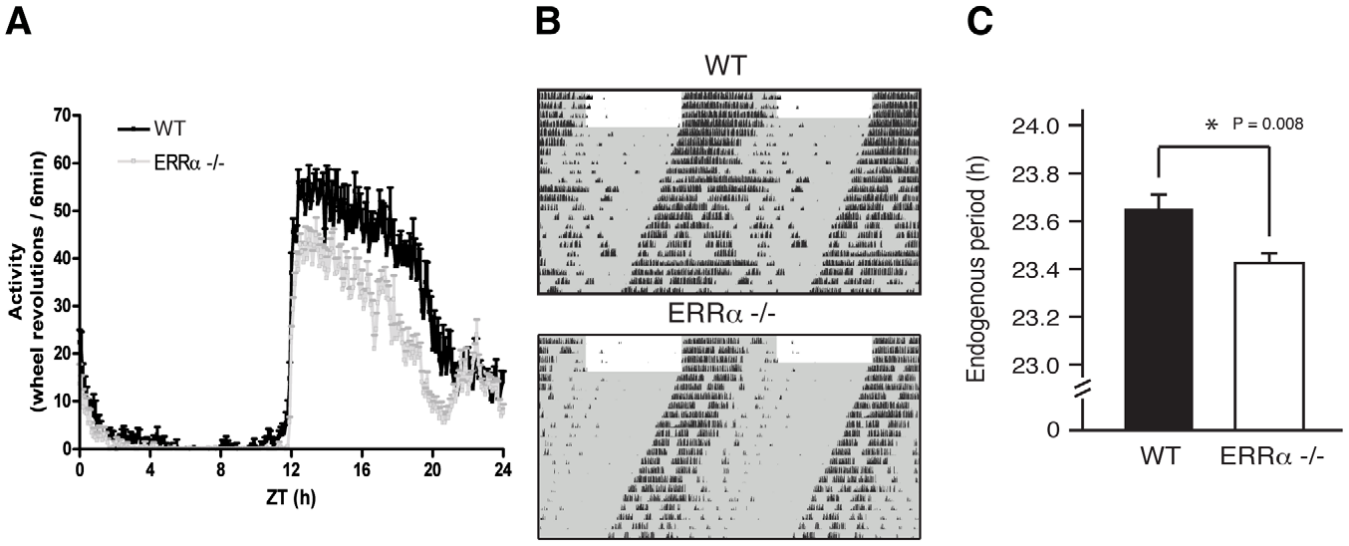
Altered locomotor activity in ERRα-null mice. (A) Locomotor activity profiles for WT and ERRα-null mice (n = 9–10) maintained in LD conditions in running wheel cages. (B) Representative actograms of one WT and one ERRα-null mouse first entrained in LD conditions and then kept in constant darkness (DD) for 3 weeks in running wheel cages. Grey zones represent darkness. (C) Bar graph representing the endogenous period lengths of WT (n = 9) and ERRα-null mice (n = 10) in constant darkness. Error bars represent ± SEM. Student's t test, *p = 0.008.

We next monitored locomotor activity in WT and ERRα-null mice. Mice in running wheel cages were first entrained in LD conditions prior to wheel-running activity recordings over a 5 day period. Under these light-entrained conditions, ERRα-null mice were found to display significantly lower activity levels, ran significantly less in the hours preceding lights off (ZT 10–12), and presented an earlier activity offset (Table 1, Figure 3A). Subsequently, the mice were put in dark/dark (DD) conditions for 20 days and the recordings from day 3 to day 20 were used to define circadian locomotor measurements in free-running conditions. Representative actograms of WT and ERRα-null mice are shown in Figure 3B. The ERRα-null mice were found to exhibit a free-running period significantly shorter than that of WT mice (23.42 hrs vs. 23.65 hrs, p = 0.008) (Table 1, Figure 3C). In addition, mice lacking ERRα displayed lower activity levels over a 24 hr period and a lower proportion of their activity in the subjective day under DD conditions (Table 1). Overall, these results implicate ERRα as a potential regulator of the circadian clock.

**Table 1:** Circadian phenotypic characterization of ERRα-null mice.

Characteristic	WT (n = 9)	ERRα-null (n = 10)	p-value Student's t test
***Activity levels***			
LD: wheel counts/24 hrs	**26995±1759**	**18206±919**	**0.0002**
DD: wheel counts/24 hrs	**27360±2879**	**21759±2188**	**0.018**
% activity (ZT 0–12)	5.8±1.2	3.5±1.1	0.164
% activity between ZT 10–12	**1.1±0.5**	**0.1±0.1**	**0.032**
% activity (CT 0–12)	**17.2±2.4**	**9.2±2.0**	**0.012**
% activity between CT 10–12	5.0±1.7	4.2±1.2	0.892
***Phase of activity in LD***			
Phase activity onset (ZT 12, min)^1^	0.0±3.0	4.8±0.6	0.064
Phase activity offset (ZT 0, min)^1^	**25.2±7.8**	**−25.8±10.8**	**0.0014**
***Running bouts***			
Average bout length (min)	169.7±13.5	162.5±13.1	0.688
Average counts/bout	**7224±814**	**5066±431**	**0.021**
Average peak speed (rotations/min)	50.4±2.1	52.2±1.9	0.507
Bouts/day	4.3±0.7	3.7±0.2	0.320
***Circadian behavior***			
Endogenous period (hrs)	**23.65±0.06**	**23.42±0.04**	**0.008**

Results in bold are statistically different between WT and ERRα-null mice.

1 Phase angle relative to lights off (ZT 12) for activity onset and relative to lights on (ZT 0) for activity offset.

### ERRα regulates the diurnal expression of core clock and clock-controlled genes in the liver

**Figure 4:**
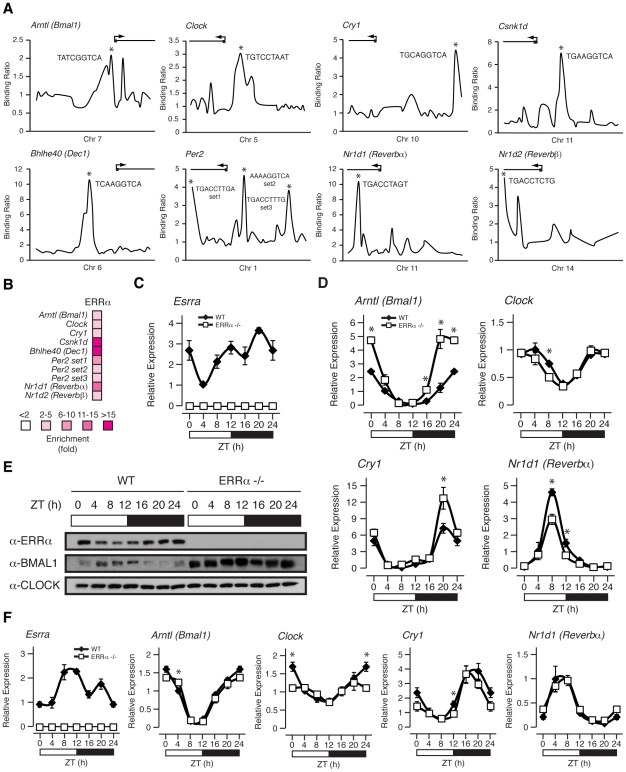
ERRα binds to and regulates core clock gene expression. (A) Binding profiles of ERRα on the extended promoters of core clock genes in mouse liver. Putative ERRα binding sequences (ERREs) are shown on the major binding peaks indicated with an asterisk. (B) Standard ChIP validation reveals that ERRα is enriched at molecular clock target genes using primers amplifying the identified binding peaks in *A*. (C–D) Male WT and ERRα-null mice (n = 4) kept in LD conditions were sacrificed at 4 hr intervals over a 24 hr period from ZT 4 to ZT 24. Circadian qRT-PCR analysis of *Esrra* (C) and molecular clock components (D) were performed on RNA isolated from mouse livers and relative expression data normalized to *Arbp* levels are shown as a function of ZT. Data shown are relative to WT expression levels at ZT 4 arbitrarily set to 1. ZT 0 values are a duplicate of ZT 24 shown for clarity. Error bars represent ± SEM. Student's t test was used to compare WT and ERRα KO liver expression data at the indicated time points, *p<0.05. (E) Western blot analysis of diurnal WT and ERRα-null mouse liver nuclear lysates. Protein extracts from 4 mice at each of the indicated time points were pooled and immunoblotted for ERRα, BMAL1 and CLOCK. (F) Male WT and ERRα-null mice (n = 4) kept in LD conditions were sacrificed at 4 hr intervals over a 24 hr period from ZT 4 to ZT 24 following a 24 hr fast. Circadian qRT-PCR analysis of *Esrra* and molecular clock components were performed on RNA isolated from mouse livers and relative expression data normalized to *Arbp* levels are shown as a function of ZT. Data shown are relative to WT expression levels at ZT 4 arbitrarily set to 1. ZT 0 values are a duplicate of ZT 24 shown for clarity. Error bars represent ± SEM. Student's t test was used to compare WT and ERRα KO fasted liver expression data at the indicated time points, *p<0.05.

We have recently shown that ERRα regulates a large number of target genes involved in a broad range of molecular functions in the liver as determined by genome-wide ChIP-on-chip analyses [34]. This dataset has not only reinforced the importance of ERRα in the control of cellular metabolism but now provides evidence for its regulation of the molecular clock. Figure 4A displays ERRα ChIP-on-chip binding profiles on the core clock genes *Arntl* (known as *Bmal1*), *Clock*, *Cry1*, *Per2*, *Nr1d1* (known as *Rev-erbα*), *Nr1d2* (known as *Rev-erbβ*), *Csnk1d* and *Bhlhe40* (known as *Dec1*). Putative ERR binding elements (ERREs) within the major binding peaks are denoted by an asterisk (Figure 4A). ChIP-qPCR validation of these binding events are shown in Figure 4B.

The gene encoding ERRα, *Esrra*, is expressed rhythmically in the liver of *ad libitum* fed mice (Figure 4C). The lowest level of *Esrra* expression was consistently observed at ZT 4 but we identified 2 peak levels at ZT 12 and ZT 20 whereas other groups identified *Esrra* expression peaks at ZT 12–16 and ZT 16 [17], [26]. The slight variability in the peak expression times of *Esrra* between laboratories may be due to differences in the housing conditions of the mice and/or exact timing and delay in tissue isolation between mice at the different time points. Overall, *Esrra* displays a rhythmic expression pattern with trough and peak expression levels at ZT 4 and ZT 12–20, respectively. Moreover, *Esrra* expression is under the control of the circadian clock as the hepatic rhythmic expression of *Esrra* under constant darkness in *Clock* mutant mice is lost [26].

We next sought to investigate whether ERRα is required to maintain the diurnal rhythm of clock gene expression in mouse liver. As shown in Figure 4D, mice lacking ERRα have altered diurnal rhythms of the clock genes *Bma1l*, *Clock*, *Cry1* and *Rev-erbα*. In all cases, a difference in expression amplitude rather than a phase-shift was observed in ERRα-null livers compared to wild-type. The livers of ERRα-null mice express a significantly higher level of *Bmal1* between ZT 16–24, less *Clock* and *Rev-erbα* levels at ZT 8 and more *Cry1* levels at ZT 20. Diurnal protein levels of ERRa, BMAL1 and CLOCK were determined by Western blot analysis as shown in Figure 4E. In wild-type mice, nuclear ERRα protein levels were found to increase during the dark cycle of the day and interestingly found to be expressed anti-phase to that of BMAL1. *Esrra* mRNA expression precedes that of *Bmal1* which helps explain the observed anti-phase diurnal protein profiles of these two factors. As expected from the mRNA profiling data, BMAL1 protein levels are significantly increased across the day in mouse liver lacking ERRα (Figure 4E). This result demonstrates that ERRα activity results in strong repression of BMAL1. In contrast, CLOCK protein levels were found to be relatively constant throughout the day in both WT and ERRα-null livers (Figure 4E). Our data provide evidence for ERRα as a direct regulator of clock gene expression in mouse liver under *ad libitum* feeding. Unexpectedly, *Esrra*, *Bmal1*, *Clock*, *Cry1* and *Rev-erbα* expression oscillate less robustly under fasting conditions and loss of ERRα expression alters diurnal clock gene expression to a much lesser extent compared to that seen in fed liver (Figure 4F).

**Figure 5:**
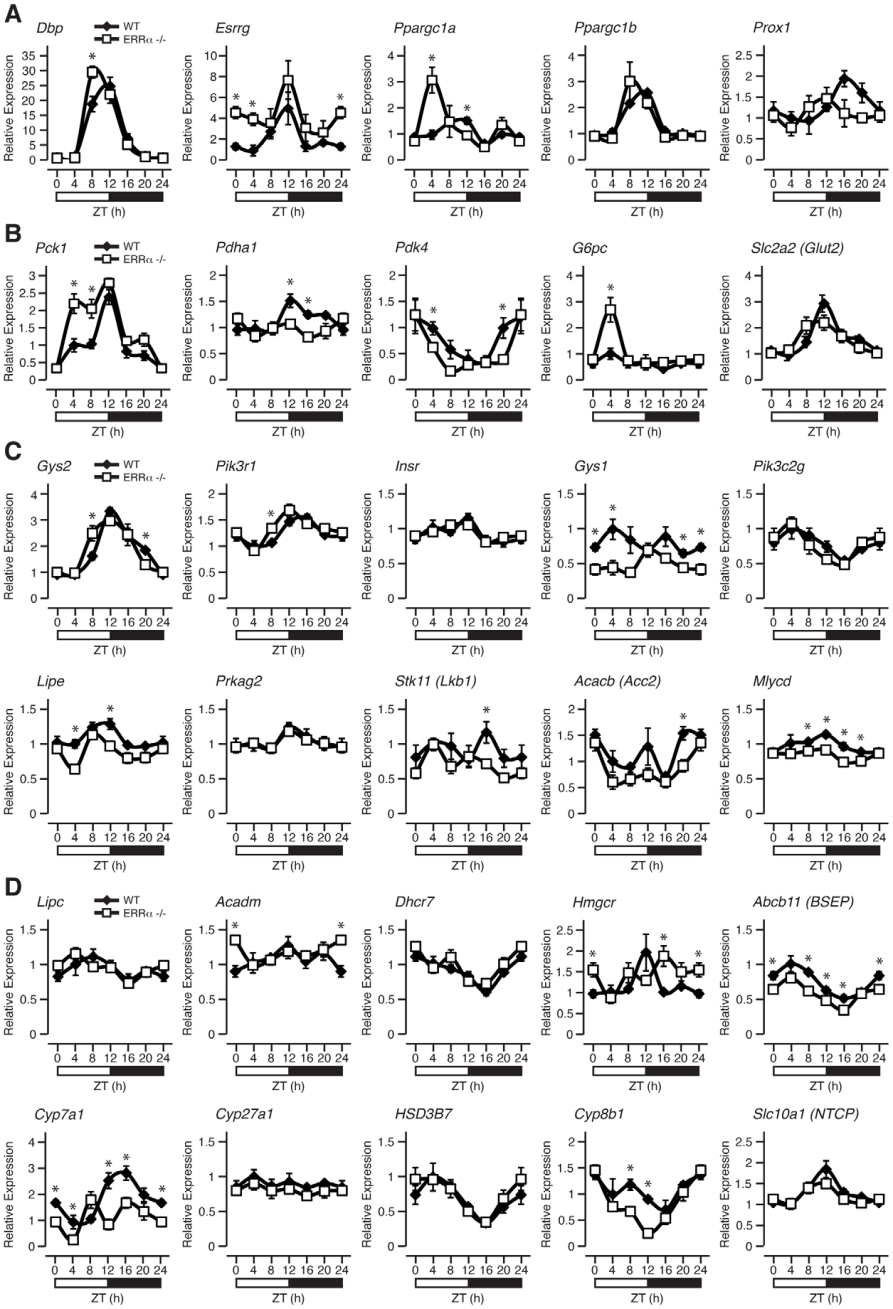
ERRα is necessary to maintain the rhythmic expression of metabolic genes. Circadian expression of genes involved in transcriptional regulation (A), glycolysis/gluconeogesesis (B), insulin and AMPK signaling (C) as well as in lipid metabolism (D) are shown. Male WT and ERRα-null mice (n = 4) kept in LD conditions were sacrificed at 4 hr intervals over a 24 hr period from ZT 4 to ZT 24. qRT-PCR analysis was performed on RNA isolated from mouse livers and relative expression data normalized to *Arbp* levels are shown as a function of ZT. Data shown are relative to WT expression levels at ZT 4 arbitrarily set to 1. ZT 0 values are a duplicate of ZT 24 shown for clarity. Error bars represent ± SEM. Student's t test was used to compare WT and ERRα KO liver expression data at the indicated time points, *p<0.05.

We next explored the importance of ERRα expression in regulating the diurnal expression of genes involved in metabolism under fed conditions as many metabolic genes are known to be under the direct transcriptional control of the receptor [34]. First, we examined the hepatic diurnal expression of transcriptional regulators known to play a role in metabolic control. As shown in Figure 5A and Figure S1A, we found altered transcript profiles of the transcription factors *Dbp*, *Esrrg* (encoding ERRγ, NR3B3), *Ppargc1a* (encoding PGC-1α) and *Nr0b2* (encoding SHP) in ERRα-null mice. No significant change in expression patterns of *Ppargc1b* (encoding PGC-1β), *Prox1* and *Nr1h4* (encoding FXR) were found (Figure 5A and Figure S1A). In addition, the levels of the mature form of the liver enriched ERRα microRNA target *miR-122a*, involved in the regulation of cholesterol and lipid metabolism [36], were found to oscillate in the liver and to display a disrupted cyclic expression in ERRα-null mice (Figure S1A). The observation that the expression of the mature form of *miR-122a* and not solely the primary transcript oscillates in liver is distinct from that of a recent report [37], but the reason for this difference is unknown. Furthermore, mature microRNA miR-378*, recently shown to act as a negative regulator of the TCA cycle and oxidative metabolism by down-regulating ERRγ and GABPA expression [38] was also found to have an altered diurnal expression in ERRα-null mice (Figure S1A). Subsequently, we examined the rhythms of genes involved in glycolysis/gluconeogenesis, insulin and AMPK signaling, lipid metabolism, the TCA cycle and OXPHOS. In ERRα-null liver, altered mRNA oscillations of the genes *Pck1*, *Pdha1*, *Pdk4*, *G6pc* and *Gck* involved in glycolysis/gluconeogenesis was observed (Figure 5B and Figure S1B). In contrast, no significant difference in expression profiles of *Slc2a2* (encoding GLUT2), *Pklr*, *Pcx* and *Pdk1* were detected. Of note, expression of the gluconeogenic genes *Pck1* and *G6pc* are significantly up-regulated in ERRα-null liver at ZT 4 during the fasting phase of the LD cycle where ERRα expression is normally at its lowest (Figure 5B). At this time point, the transcript encoding PGC-1α, a known activator of the gluconeogenic transcriptional program, is also drastically increased in the absence of ERRα (Figure 5A). Our data thus validate the previous report demonstrating that ERRα acts as a repressor of gluconeogenesis in contrast to the overall positive action of PGC-1α in this process [39]. Next, we analyzed genes associated with insulin and AMPK signaling. Briefly, ERRα-null livers express altered diurnal rhythms of the genes *Gys2*, *Pik3r1*, *Gys1*, *Lipe*, *Stk11* (encoding LKB1), *Acacb* (encoding ACC2) and *Mlycd* with no significant change in the mRNA profiles of *Insr*, *Pik3c2g* and *Prkag2* (Figure 5C). In Figure 5D, we demonstrate that the diurnal expression patterns of genes involved in fatty acid, cholesterol and bile acid metabolism are dependent on ERRα. Specifically, *Acadm*, encoding the enzyme MCAD important in fatty acid β-oxidation [40] and *Hmgcr*, encoding the rate-limiting enzyme HMG-CoA reductase in cholesterol biosynthesis [41] are up-regulated in ERRα-null mice at specific times during the day. Despite the increased expression of *Hmgcr* found in the liver, ERRα-null mice have reduced circulating levels of cholesterol during the light phase of the day as shown earlier. Moreover, hepatic genes involved in bile acid biosynthesis and transport *Cyp7a1*, *Cyp8b1* and *Abcb11* (encoding BSEP) respectively, are generally down-regulated in the absence of ERRα (Figure 5D). Consequently, decreased circulating bile acids would be anticipated in ERRα-null mice but, as described above, we found increased levels at ZT 0/24. Reduced *Cyp7a1* and *Cyp8b1* gene expression in ERRα-null liver may be attributable to possible negative feedback inhibition exerted by the higher bile acid levels but the exact mechanism is unknown. The expression profiles of genes associated with mitochondrial energy production were also explored. Overall, we found a significant reduction in the expression profiles across a 24 hr cycle of many TCA cycle and OXPHOS genes including *Cs*, *Aco2*, *Sdhd*, *Cycs*, and *Ndufb5* in ERRα-null liver (Figure S1C). Taken together, our data clearly defines ERRα as an important player in the diurnal regulation of clock gene expression and of many metabolic genes involved in clock-controlled physiological outputs. Notably, oscillations of several metabolic genes that are not known to be direct targets of ERRα were altered in the liver including *Esrrg*, *Ppargc1a* and *Cyp7a1*. Our data suggest that ERRα participation in the maintenance of cyclic gene expression is mediated by direct and indirect transcriptional control of genes via regulation of the molecular clock and other transcriptional regulators.

### Genomic convergence between BMAL1/CLOCK and ERRα in the transcriptional control of metabolic genes

**Figure 6:**
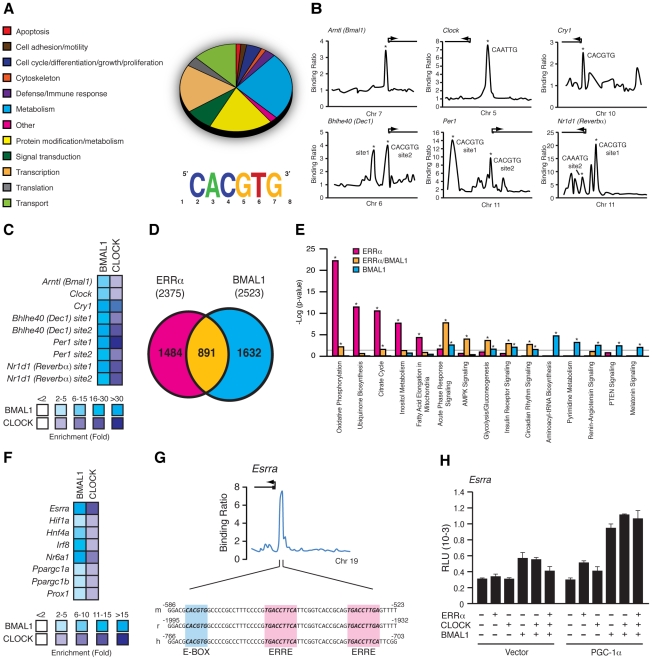
BMAL1/CLOCK regulation of the ERRα promoter. (A) Pie chart representing the major cellular functions associated with BMAL1 targets enriched in the adult mouse liver using extended promoter arrays. Motif finding algorithm, MDscan, showed that the sequence CACGTG corresponding to the consensus BMAL1/CLOCK E-box binding motif is the most abundant motif present in the first 200 BMAL1 bound segments used in the analysis. (B) Binding profiles of BMAL1 on extended promoters of a subset of core clock gene targets obtained from ChIP-on-chip. Putative BMAL1/CLOCK binding sequences (E-boxes) found, if any, at the binding peaks validated indicated with an asterisk are shown. (C) BMAL1/CLOCK standard ChIP assay in mouse liver on the core clock genes shown in *B*. (D) Venn Diagram illustrating the overlap in ERRα and BMAL1 direct targets obtained from ChIP-on-chip analyses in mouse liver. (E) Enrichment of a subset of canonical pathways in the ChIP-on-chip target genes determined to be common (yellow) or specific to either ERRα (pink) or BMAL1 (blue). Grey line indicates the p-value threshold cutoff of 0.05. *p<0.05. (F) BMAL1/CLOCK standard ChIP assay in mouse liver on genes associated with transcriptional regulation including ERRα. (G) Schematic representation of a fragment of the mouse, rat and human ERRα promoter showing a conserved BMAL1/CLOCK E-box consensus binding motif, CACGTG, adjacent to the conserved ERRα binding element, TGAAGGTCA. (H) Reporter gene assays in COS-1 cells using a mouse *Esrra* promoter luciferase reporter construct in the presence of either empty vector, ERRα, CLOCK, BMAL1 or combinations of in the presence or absence of PGC-1α. Luciferase activity was assayed 24 hrs post-transfection and the relative luciferase units (RLU) shown are the mean of duplicate assays.

We next sought to determine the extent of the functional relationship between BMAL1 and ERRα in the direct control of metabolic gene networks. To this end, we first performed a mouse liver BMAL1 ChIP-on-chip experiment using tiled arrays covering extended promoter regions (−5.5 to +2.5 kb from transcriptional start sites) of ∼17,000 genes, the same platform previously used to identify ERRα occupancy in the genome of mouse livers taken at ZT 4 in LD conditions [34]. We identified 2,555 high-confidence BMAL1 binding sites mapping to the promoter regions of 2,522 genes (Table S1). First, we classified the target genes associated with a known function into general cellular functional categories as shown in Figure 6A. BMAL1 was found significantly enriched at promoters of genes involved in a broad range of metabolic processes, including amino acid, lipid, carbohydrate and TCA cycle/OXPHOS. In addition, ∼15% of the BMAL1 target genes are involved in transcriptional regulation, including genes encoding numerous transcription factors, nuclear receptors involved in metabolic control (see below), splicing factors and polymerases. *De novo* motif discovery using MDscan [42] analysis of enriched binding segments revealed the expected consensus E-box motif, CACGTG (Figure 6A). BMAL1 enrichment at the extended promoter regions of core clock genes including *Cry2*, *Dbp*, *Dec1*, *Dec2*, *Per1*, *Per2*, *Per3* and *Rev-erbα* further validates the approach using extended promoter arrays (Figure 6B and Figure S2). Furthermore, our analysis revealed previously unidentified binding sites for BMAL1/CLOCK within the promoters of *Bmal1* and *Clock* themselves as well as in the *Csnk1d* promoter (Figure 6B and Figure S2). *Cry1*, a known BMAL1/CLOCK target gene, missed the ChIP-on-chip p-value cutoff but did validate in standard BMAL1 and CLOCK ChIP-qPCR experiments along with other tested target genes (Figure 6C). Our data not only shows that BMAL1/CLOCK can directly bind to the core clock genes but that they can also directly bind to their own promoters, thus identifying a previously unrecognized autoregulatory loop.

We next compared the overlap in target genes between ERRα and BMAL1 in mouse liver. As shown in Figure 6D, comparison of the datasets revealed that a total of 891 target genes are shared by both factors (37.5% of all ERRα targets). A significant number of common targets, which include *Pck1*, *Hmgcr*, *Nr0b2* and *miR-122a*, were found to have altered cyclic expression patterns in ERRα-null mice (Figure 5 and Figure S1), amplifying the importance of ERRα as a transcriptional regulator of clock-controlled genes. We next identified significantly enriched biological pathways associated with target genes that are specific to ERRα, BMAL1 or both factors. A subset of the analysis is shown in Figure 6E. ERRα-specific targets were highly enriched in metabolic and energy producing processes including inositol metabolism, lipid metabolism, the TCA cycle, ubiquinone biosynthesis and OXPHOS. Targets shared by ERRα and BMAL1 were also associated with metabolic processes such as AMPK and insulin receptor signaling in addition to glycolysis/gluconeogenesis. ChIP qPCR validation of ERRα and BMAL1 enrichment at the promoters of several genes involved in these processes including *Stk11*, *Prkag2*, *Insr*, *Pik3c2g*, *Gys2* and *G6pc* is shown in Figure S3. Moreover, there was a strong enrichment of common ERRα and BMAL1 target genes involved in immune response as well as in circadian rhythm signaling (Figure 6E). Targets specific to BMAL1 were enriched in a wide variety of processes associated with protein assembly, nucleic acid metabolism as well as renin-angiotensin, hormone and cancer signaling (Figure 6E). In order to execute clock-controlled physiological and behavioral processes in a timely and efficient manner, BMAL1/CLOCK regulate a number of factors involved in transcription to mediate clock outputs. As we previously noted, BMAL1 was found to be enriched at over 300 target genes associated with transcriptional regulation including genes encoding the nuclear receptors ERRα, COUP-TFII (NR2F2), GCNF (NR6A1), HNF4α (NR2A1), LRH-1 (NR5A2), PPARa PPARγ (NR1C3), PXR (NR1I2), SHP (NR0B2), REV-ERBα, RORγ, RXRβ (NR2B2), SF-1 (NR5A1) and TRα, the transcription factors ATF2-7, DBP, HIF1α, IRF8, GABPA, and STAT3, and the transcriptional coregulators PGC-1α, PGC-1β, NCoR1 and PROX1. In addition, the microRNAs miR-17, -22, -101a, -122a, -200c and let-7c-2 were also identified as BMAL1 targets. BMAL1/CLOCK ChIP validation of a subset of these genes is shown in Figure 6F. In particular, our data show that ERRα is indeed a direct downstream target of BMAL1/CLOCK. Figure 6G displays the binding profile of BMAL1 on the mouse *Esrra* promoter with close examination of the DNA sequence under the peak. We identified a BMAL1/CLOCK consensus E-box binding motif (CACGTG) located adjacent to the well characterized, duplicated ERR binding sites (TNAAGGTCA) [29] that is conserved in mouse and human (Figure 6G). Luciferase reporter assays in COS-1 cells show that BMAL1 alone induces the activity of the *Esrra* promoter which was further enhanced in the presence of PGC-1α (Figure 6H).

### Prox1 expression is rhythmic and regulates clock gene expression

**Figure 7:**
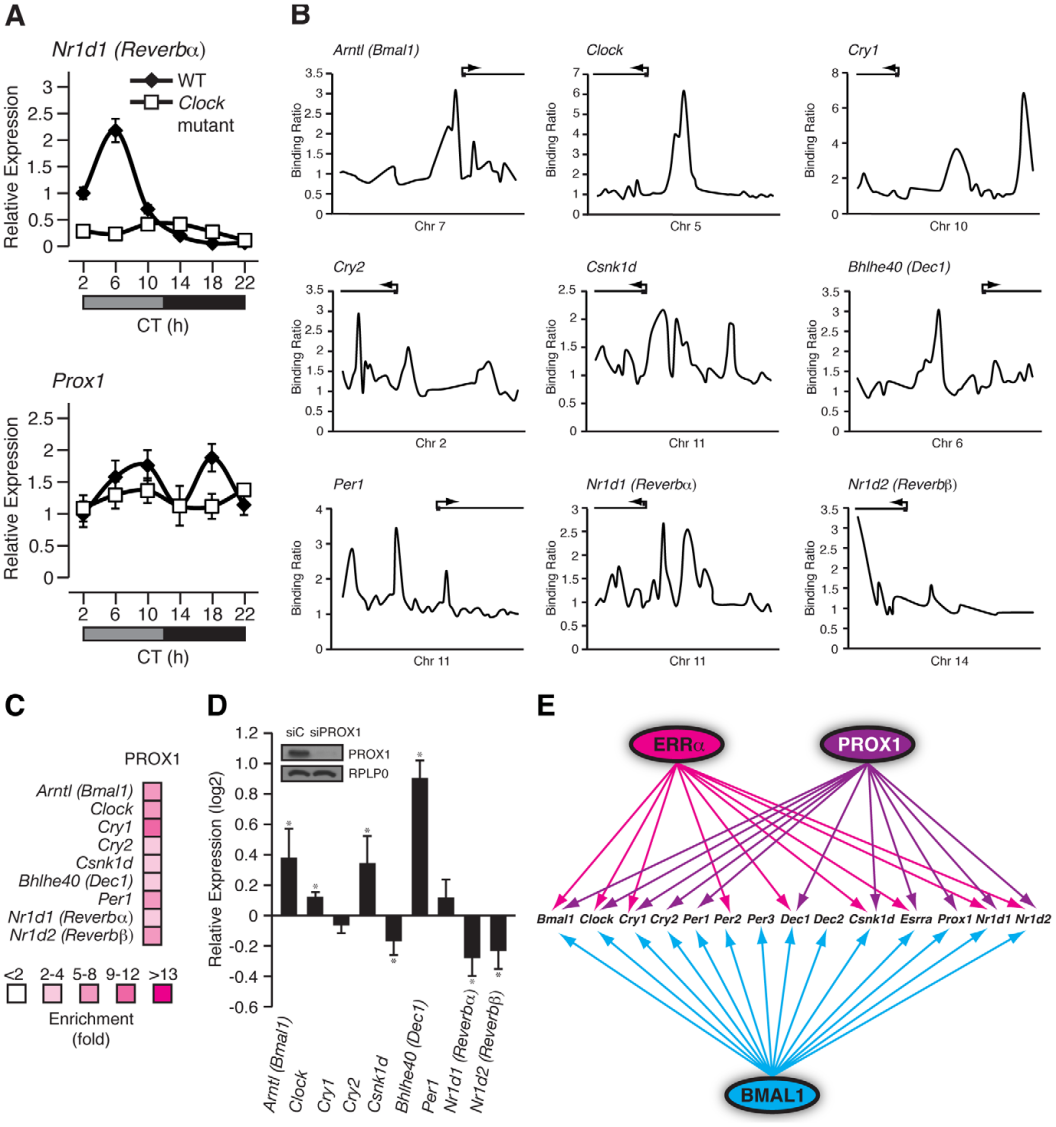
PROX1 is a regulator of the molecular clock that itself exhibits a rhythmic clock-dependent circadian expression. (A) Male WT and *Clock* mutant mice (n = 4) kept in DD conditions were sacrificed every 4 hrs over a 24 hr period from circadian time (CT) 2 to CT 22. qRT-PCR analysis was performed on RNA isolated from mouse livers and relative expression data normalized to *Arbp* levels are shown as a function of CT. Error bars represent ± SEM. (B) Binding profiles of PROX1 on the extended promoters of core clock genes generated from a recently published ChIP-on-chip experiment in mouse liver. (C) Standard ChIP validation of molecular clock genes identified as direct target genes of PROX1. (D) qRT-PCR was performed on RNA isolated from HepG2 cells treated with control siRNA or an siRNA Dharmacon On-Target Smartpool against PROX1. The expression of molecular clock genes was determined and the data is shown as relative fold expression levels compared to control siRNA and normalized to *HPRT1* levels. Data represent mean ± s.d. of triplicate independent experiments. Student's t test, *p<0.05. Western blot analysis on lysates prepared from the HepG2 knockdown samples is shown with the respective antibodies as indicated. (E) Schematic showing the transcriptional crosstalk between ERRα, PROX1 and BMAL1 on molecular clock genes as well as *Esrra* and *Prox1*. Genes found to be direct targets of ERRα, PROX1 and/or BMAL1 are indicated by arrows.

*Prox1* is a direct BMAL1/CLOCK target gene (Figure 6F) and consequently, we next sought to examine if *Prox1* expression, like that of other components of the ERRPGC-1 transcriptional pathway, is rhythmic in mouse liver. Wild-type and *Clock* mutant mice kept in constant darkness were used to measure the circadian expression of *Prox1*. As expected, *Rev-erbα* was found to have an expression peak at circadian time (CT) 6 and the mRNA oscillation was abrogated in *Clock* mutant mice (Figure 7A, top panel). Interestingly, we observed a cyclic expression in *Prox1* transcript levels with two expression peaks at CT 10 and CT 18 that was abolished in *Clock*-mutant mice under DD conditions (Figure 7A, bottom panel). Unlike in constant darkness, *Prox1* oscillates with one expression peak during the night in light-entrained conditions as shown earlier (Figure 5A) suggesting that *Prox1* expression is influenced by light. Taken together, our data indicate that *Prox1* expression is rhythmic and under direct control of the molecular clock.

We next sought to determine if PROX1 was also involved in a regulatory loop with BMAL1/CLOCK. Indeed, ChIP-on-chip experiments revealed PROX1 recruitment to the clock target genes *Bmal1*, *Clock*, *Cry1*, *Cry2*, *Csnk1d*, *Dec1*, *Per1*, *Rev-erbα* and *Rev-erbβ* as shown in Figure 7B. ChIP-qPCR validation of these binding events are shown in Figure 7C. We next wanted to investigate PROX1 regulation of the molecular clock. Unlike ERRα-null mice, PROX1-deficient mice are embryonic lethal and die at embryonic day E14.5-E15 [43]. Consequently, we used the HepG2 liver cell line to analyze the expression of direct clock target genes in the presence or absence of siRNA pools targeting PROX1. Ablation of PROX1 in HepG2 cells resulted in an increase in *Bmal1, Clock, Cry2 and Dec1* expression as well as a decrease in *Csnk1d*, *Rev-erbα* and *Rev-erbβ* levels (Figure 7D). Subsequently, serum shock was used to synchronize clock gene oscillation in HepG2 cells to determine the effects of loss of PROX1 on the rhythmic expression of these genes. qRT-PCR analysis of clock gene expression over a 24 hr period was studied. Overall, ablation of PROX1 in synchronized HepG2 cells resulted in significantly altered oscillations and abundances of clock transcripts (Figure S4A). Our data show that PROX1 is a regulator of the molecular clock by acting as either an activator or repressor of clock gene expression. Moreover, we observed altered expression rhythms of *Pck1*, *Slc2a2* and *Aldoc* involved in glucose homeostasis in the absence of PROX1 in synchronized HepG2 cells (Figure S4B). As a whole, PROX1 can function both as an upstream clock regulator and direct downstream mediator of clock function. A schematic representation of the integration of ERRα and PROX1 as well as BMAL1 in transcriptional control of mammalian clock components is shown in Figure 7E.

### Convergence of ERRα, PROX1, and BMAL1 target genes integrates the molecular clock with metabolism

**Figure 8:**
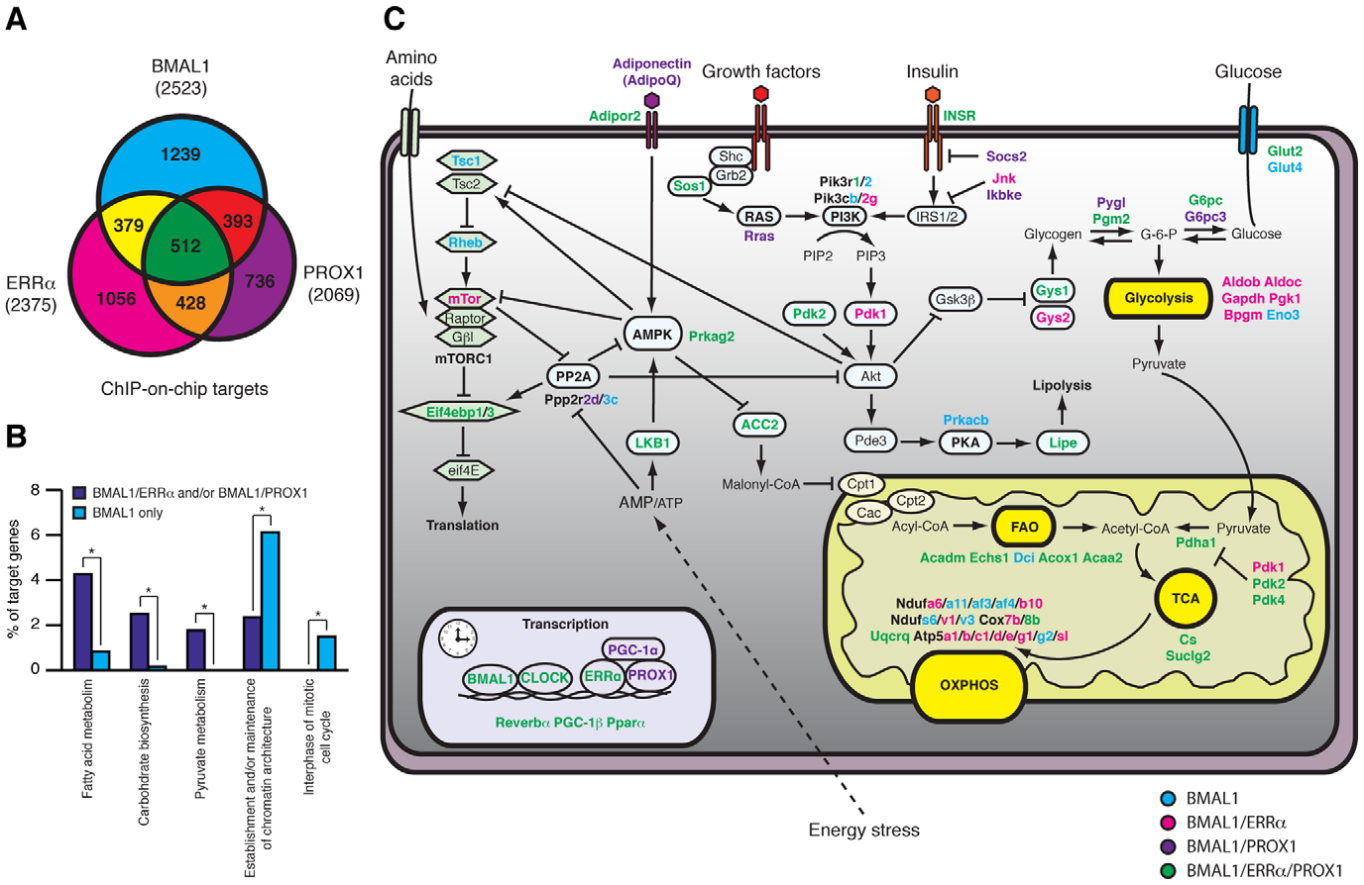
ERRα, PROX1, and BMAL1 genomic convergence linking the clock with metabolism. (A) Venn Diagram illustrating the overlap in ERRα, PROX1 and BMAL1 direct targets obtained from ChIP-on-chip analyses in mouse liver. (B) Comparison between the functionally enriched biological processes associated with BMAL1 targets shared by ERRα and/or Prox1 or bound by BMAL1 only. Target genes specific for BMAL1 association with ERRα and/or Prox1 were enriched for metabolic processes where targets specific to BMAL1 alone were enriched for processes related to chromatin structure and cell cycle. *p<0.01. (C) Schematic representing target genes specific to BMAL1 (blue) or those shared by BMAL1/ERRα (pink), BMAL1/PROX1 (purple) or BMAL1/ERRα/PROX1 (green) associated with diverse metabolic processes.

Analysis of the gene networks commonly regulated by ERRα, PROX1 and/or BMAL1 shows that 905 (∼35.8% of all BMAL1 targets) target genes are common to PROX1 and BMAL1 and that 891 (∼35.3% of all BMAL1 targets) target genes are shared by ERRα and BMAL1 (Figure 8A and Table S2). Overall, 512 targets are shared by all 3 factors (∼20% of all 3 datasets), indicating a significant level of coordination in the control of specific gene networks. Comparative analysis of these datasets with mouse liver circadian expression data compiled from five different experiments performed with mice under basal conditions maintained in constant darkness [12], [44]–[47] indicates that a large subset of ERRα, BMAL1 and PROX1 target genes displays rhythmic expression in the liver (Figure S5 and Table S3). A gene found to be rhythmic in at least one dataset was considered in our analysis. Interestingly, we also observed that genes commonly targeted by either BMAL1 and ERRα, BMAL1 and PROX1 or by all three factors are enriched for metabolic genes (e.g. fatty acid and carbohydrate metabolism) as compared to genes targeted by BMAL1 alone (Figure 8B). A schematic representation of the metabolic and nutrient sensing pathways targeted by BMAL1 alone or in combination with ERRα and PROX1 is shown in Figure 8C.

## Discussion

In this study we first identified ERRα, an orphan nuclear receptor known to play a central role in the control of energy homeostasis, as an important regulator of the mammalian circadian clock and its output pathways at both transcriptional and physiological levels. Indeed, we have shown that ERRα occupies the promoter region of several core clock genes and that *Esrra* is itself a direct transcriptional target of BMAL1/CLOCK. While control of the expression of transcription factors involved in metabolic homeostasis provides a sensible molecular mechanism linking core clock genes and metabolism, our results also revealed that BMAL1 directly targets an unexpectedly large number of genes associated with a wide-array of biological processes, including cellular metabolism. In fact, the extensive transcriptional crosstalk between ERRα, BMAL1 and the metabolic coregulator PROX1 firmly positions these three factors at the center of the coordinated control of circadian rhythms and cellular metabolism (Figure 8C).

### ERRα, a regulator of diurnal metabolic homeostasis

As a direct target of BMAL1/CLOCK and a major regulator of energy metabolism, ERRα may also play a central role in clock-controlled output pathways linked to metabolic homeostasis. Indeed, the importance of ERRα as an integrator of the molecular clock and metabolism is evident *in vivo* as the absence of ERRα in mice results in altered hepatic diurnal expression rhythms of genes associated with diverse metabolic processes, the majority of which are known to display circadian rhythms (e.g. *Cyp7a1*, *Hmgcr*, *Pck1*, *Pdk4*, *PGC-1α* and *Stk11*). In this context, the finding of the potential regulation of *Per2* by ERRα is also particularly interesting. PER2 has recently been shown to interact with several nuclear receptors and serve as a coregulator of nuclear receptor-mediated transcription and it has thus been proposed that PER2 could confer more precise oscillator information to metabolic genes [23]. Given that ERRα has been shown to occupy the regulatory region of genes encoding a plethora of nuclear receptors that includes the PER2 partners HNF4α, PPARα and REV-ERBα [34], ERRα could serve as an amplifier of the PER2-dependent clock output.

Diurnal serum chemistry profiling revealed an important role of ERRα in maintaining metabolic homeostasis. Altered diurnal glucose, insulin, bile acid, cholesterol, NEFA and triglyceride levels were observed in ERRα-null mice. Of particular interest, fed ERRα-null mice exhibit time-dependent hypoglycemia and hypoinsulinemia with no apparent impairment in insulin secretion as determined by glucose tolerance tests. We thus hypothesize that the observed hypoglycemia in ERRα-null mice is a result of enhanced glucose uptake due to increased insulin sensitivity. In complete agreement with our findings in ERRα-null mice, a report published during preparation of this manuscript showed that administration of a novel highly selective ERRα inverse agonist (compound 29) in diet-induced murine models of obesity and an overt diabetic rat model resulted in improved insulin sensitivity and glucose tolerance accompanied by reduced circulating glucose, free fatty acid and triglyceride levels [48].

### ERRα, a novel direct regulator of the circadian clock

Nuclear receptors have been previously implicated as components of the output pathways of the molecular clock but their involvement in these processes has usually been inferred through the regulation of one to a few specific genes [22]. Previous *in situ* hybridization experiments revealed that 19 of 49 nuclear receptors are expressed in the mouse SCN [49]. However, lack of apparent expression of any ERR isoform in the SCN suggests that the ERRs are not critically involved in SCN physiology and clock entrainment. This is supported by the modest change in free-running period of locomotor activity rhythms (Figure 3). Instead, ERRs may act as regulators of clock function in peripheral tissues. Indeed, this study demonstrates that ERRα plays a key role in hepatic regulation of the molecular clock.

We show that ERRα, as a regulator of the core clock mechanism, targets the promoter region of many core clock genes, including *Bmal1*, *Clock*, *Cry1*, *Per2*, *Rev-erbα* and *Rev-erbβ* and that the presence of ERRα is necessary to maintain the diurnal rhythm of many of these genes in the liver. Of particular interest, ERRα was found to display very potent repressor activity on BMAL1 (Figure 4D and 4E). Unlike the nuclear receptor REV-ERBα that couples the positive and negative limbs of the molecular oscillator, the role of ERRα in the clock is more likely to contribute to the robustness of circadian oscillation in response to specific physiological cues. In this context, physiological rhythms dependent on ERRα transcriptional activity is expected to be modulated via changes in the levels of PGC-1α in response to nutritional signals [33], and/or the activity of the SIRT1 histone deacetylase complex which is known to act as an intracellular metabolic sensor and a post-translational modifier of both PGC-1α and ERRα [50], [51]. This is particularly relevant in regard to the superimposed metabolic and circadian clock feedback loops involving interplay between the rhythmic NAD^+^ biosynthesis, SIRT1, and CLOCK/BMAL1 [52], [53].

### A direct role for BMAL1 in liver metabolism

BMAL1 is an important regulator of metabolism. In particular, liver-specific disruption of *Bmal1* in mice results in hypoglycemia, higher glucose clearance and loss of rhythmic expression of clock-regulated metabolic genes, highlighting the importance of the peripheral oscillators in modulating circadian physiology [14], [52]. Our BMAL1 ChIP-on-chip study in mouse liver indicates that BMAL1 plays a more comprehensive role in dictating metabolic clock outputs. We find that BMAL1 binding sites are particularly enriched in genes involved in amino acid (e.g. *Got1*, *mTor*), lipid (e.g. *Acadm*, *Lipe*) and carbohydrate (e.g. *G6pc*, *Insr*) metabolism as well as in the TCA cycle/OXPHOS (e.g. *Cs*, *Atp5g*) (Figure 8C and Table S1). Of particular interest is the finding that BMAL1 binds to two distinct sites within the promoter of *Slc2a2*, which encodes glucose transporter type 2 (GLUT2). This finding thus provides a molecular mechanism for the loss of rhythmic expression of *Slc2a2* in liver-specific BMAL1-deficient mice which has been proposed to account for the observed circadian hypoglycemia in these mutant mice [14]. More globally however, our study demonstrates that direct BMAL1 target genes are not exclusively associated with metabolism but with a large set of diverse biological functions. This finding is in agreement with previous expression studies showing that temporal hepatic gene regulation is extensive and impinges on a wide variety of processes [12], [45], [46]. Our work suggests that the extensive identification of BMAL1 target genes supports a more direct participation of core clock proteins in driving clock output pathways.

### Prox1, a novel regulator of the molecular clock and metabolism

Our study provides evidence for a direct participation of PROX1 in transcriptional regulation of the molecular clock. In particular, clock gene synchronization in HepG2 cells revealed that ablation of PROX1 results in altered oscillation of core clock genes. In this context, we also show that PROX1 is required to maintain the rhythmic expression of genes involved in glucose homeostasis. Taken together with the recent identification of PROX1 as an important regulator of the ERRα/PGC-1α axis involved in the regulation of broad transcriptional programs implicated in the control of energy homeostasis in the liver [34] and the observation that the *PROX1* locus is associated with fasting glucose levels and increased risk for type II diabetes [35], this study implies that PROX1 possesses all the necessary attributes to be an important factor linking metabolism and circadian rhythms.

### Convergence of BMAL1, ERRα, and PROX1 in the rhythmic control of metabolic genes

We have shown that BMAL1 targets the promoter region of a substantial number of metabolic genes. However, a comparative analysis of BMAL1, ERRα and PROX1 targets indicates that the presence of BMAL1 alone is insufficient to regulate the expression of metabolic gene networks (Figure 8B and 8C and Table S2). In contrast, these results suggest that the regulation of metabolic genes involves coordinated action by the three factors. Whether BMAL1 can directly interact with ERRα and/or PROX1 is currently unknown. We suggest that ERRα may function by contributing to the robustness of the rhythmic expression of BMAL1 target genes. Similarly, the overlap between BMAL1 and PROX1 target genes, especially those not shared by ERRα, probably denotes the presence and action of other partners of PROX1 such as the metabolic regulators HNF-4α and LRH-1 [54], [55]. The physiological significance of the functional interaction between these factors is further supported by the observation that a considerable subset of ERRα/BMAL1/PROX1 target genes displays circadian expression in the liver (Figure S5 and Table S3). Finally, as ERRα expression is regulated by metabolic cues, we can anticipate that the rhythmic expression of ERRα and many of its target genes will be driven by both feeding and the clock.

### Summary

We have identified the orphan nuclear receptor ERRα as a novel transcriptional regulator of both the molecular clock and its output pathways that shares extensive transcriptional cross-talk with the core clock protein BMAL1. As such and given the known property of ERRα to translate signals propagated by physiological sensors such as PGC-1α and SIRT1 into metabolic gene expression networks, ERRα may serve as the key bidirectional regulator connecting the peripheral liver clock and cellular energy metabolism. Similarly, we show that the ERRα corepressor PROX1 can act both upstream and downstream of the endogenous clock. Furthermore, our study showed that the direct participation of BMAL1 in the clock output pathways is highly extensive, suggesting that other core clock proteins might play a similar role. Therefore, additional investigations of the functional relationship between ERRα, PROX1 and core clock genes in diverse tissues are bound to reveal other key molecular mechanisms and physiological phenotypes linked to the daily timing of biological processes.

## Materials and Methods

### Ethics statement

Animal use followed the guidelines of the Canadian Council on Animal Care. The animal use protocol was approved by the local Facility Animal Care Committee (FACC) at McGill University.

### Animals

Male wild-type and ERRα-null mice [56] 2–3 months old in a C57BL/6J genetic background were housed and fed standard chow in the animal facility at McGill University Life Sciences Complex. Mice were entrained to a 12 hr light/12 hr dark LD cycle for 2 weeks prior to the start of the experiment. Animals were sacrificed by cervical dislocation at 4 hr intervals over a 24 hr period from ZT 4 to ZT 24 (n = 4 per ZT). ZT 0 is the time of lights on, ZT 12 is the time of lights off. Livers were isolated, frozen in liquid nitrogen and grinded using a mortar and pestle and kept frozen until further processing. Male wild-type and *Clock* mutant mice [57] 2–3 months old in a 50% C57BL/6J and 50% BALB/c genetic background were housed and fed standard chow in the animal facility at the Douglas Mental Health University Institute. For circadian experiments, livers from 4 WT and *Clock* mutant mice kept in DD conditions were collected every 4 hrs from circadian time (CT) 2 to CT 22 and immediately frozen in liquid nitrogen until further processing.

### ChIP, ChIP-on-chip, ChIP-qPCR, and functional analysis of target genes

ERRα and PROX1 ChIP assays were performed as previously described [34], [58] on adult male mouse livers at ZT 4. For BMAL1 and CLOCK ChIP assays, chromatin corresponding to 0.4 g of initial liver mass taken from a pool of 44 livers at ZT 4 was used and pre-cleared chromatin was immunoprecipitated with 6 µg of an anti-BMAL1 antibody (Santa Cruz, sc-48790x), 6 µg of an anti-CLOCK antibody (Santa Cruz, sc-25361x) or not (no antibody control) with subsequent addition of 50 µl of a 50% slurry of salmon sperm DNA/protein A beads for 3 hrs at 4°C. To assess the enrichment of ERRα, PROX1, BMAL1 or CLOCK at specific promoters, quantitative PCR (qPCR) was performed as described previously [58]. Enrichment of DNA fragments was normalized against two amplified regions using the control primers, located approximately 4 kb upstream of the ERRα and 49 kb upstream of the PROX1 transcriptional start site. Specific mouse primers designed and used for ChIP-qPCR analysis are shown in Tables S4 and S5.

Duplicate mouse liver BMAL1 ChIP-on-chip experiments using tiled extended promoter arrays covering −5.5 to +2.5 kb from transcriptional start sites of ∼17,000 genes (mm8) from Agilent were performed as previously described [34], [58] with the following modifications. Chromatin corresponding to 3.1 g of initial liver mass taken from a pool of 44 livers was used and pre-cleared chromatin was immunoprecipitated with 45 µg of an anti-BMAL1 antibody (sc-48790x) or not (no antibody control) with subsequent addition of 400 µl of a 50% slurry of salmon sperm DNA/protein A beads for 3 hrs at 4°C. The ChIP-on-chip target genes were classified by biological function based on GO annotation (http://fatigo.org/) and NCBI gene descriptions. The mouse liver BMAL1 ChIP-on-chip bed file can be found in Dataset S1.

### Ingenuity pathway analysis of target genes

Analysis of the ChIP-on-chip target genes for significant biological pathways and networks were done using Ingenuity Pathways Analysis software v7.6 (Ingenuity Systems, www.ingenuity.com). Canonical pathways analysis identified significant pathways from the Ingenuity's Pathways Analysis library of canonical pathways. Fisher's exact test was used to calculate a p-value determining the probability that the association between the genes in a dataset and the canonical pathway is explained by chance alone. Networks of genes were algorithmically generated based on their connectivity by overlaying the target genes onto a global molecular network developed from information contained in the Ingenuity Pathways Knowledge Base.

### Constructs

pCMX, pCMX-hERRα and mESRRA-luciferase were described previously [29]. The expression vector pcDNA3/HA-hPGC-1α was provided by A. Kralli (La Jolla, CA). The expression vectors pSG5-mCLOCK and pCS2 (5× Myc)-mBMAL1 were described previously [59].

### Reporter assays

Cos-1 or HepG2 cells were transfected using FUGENE in 12-well plates with 300 ng luciferase reporter, 100 ng CMX expression vector (empty vector or hERRα), 100 ng CMV β-galactosidase, 200 ng pSG5 expression vector (empty vector or mCLOCK), 200 ng pCS2 (5× myc) expression vector (empty vector or mBMAL1), with 300 ng of HA-PGC1α or pcDNA3. Cells were harvested and assayed for luciferase activity 24 hrs post-transfection. Experiments were performed in triplicate and each experiment was replicated multiple times.

### RNA interference

HepG2 cells were cultured in DMEM (Invitrogen) supplemented with 10% FBS and pen/strep and maintained at 70% confluency. HepG2 were transfected with either On-Target Smartpool control (siCtrl) from Dharmacon or a specific siRNA pool against PROX1 (siProx1) using HiPerfect reagent (according to the manufacturer's instructions).

### Serum shock

HepG2 cells treated with either control siRNA (siCtrl) or siProx1 were grown and maintained in high glucose (25 mM) Dulbecco's modified Eagle's medium (DMEM) supplemented with 10% fetal bovine serum (FBS). 48 hrs later, the cells were then starved in DMEM containing 0.5% fetal bovine serum for 24 hrs. Subsequently, 50% horse serum was added (T = 0) for 2 hrs, and then the medium was changed back to starvation medium. RNA was isolated from cells harvested every 4 hrs during a 24 hr period for qRT-PCR analysis.

### Quantitative reverse-transcription PCR

For quantitative reverse-transcription PCR, cDNA was prepared from total RNA isolated from mouse livers and the HepG2 siRNA knock-down samples. cDNA was obtained from 2 µg of total RNA by reverse transcription with Oligo(dT) primer, dNTPs, 5× 1^st^ strand buffer, DTT, RNase inhibitor, and Superscript II RNase H Reverse Transcriptase. cDNA was amplified using specific primers (Tables S6 and S7) along with the SYBR PCR Master Mix (Qiagen) and a LightCycler instrument (Roche). Relative fold expression levels of the analyzed genes in mouse livers were normalized to *Arbp* levels and expressed as mean values +/− SEM at the indicated time points relative to the mean value of the WT livers at ZT 4 set at 1. For all genes tested, primer efficiencies were taken into account based on a serial dilution of cDNA.

Relative fold expression levels of the analyzed genes in serum shocked HepG2 cells were normalized to *HPRT1* levels and expressed as mean values +/− s.d. at the indicated time points relative to the mean value of siCtrl samples at T = 0 set at 1. For all genes tested, primer efficiencies were taken into account based on a serial dilution of cDNA.

For microRNA quantification, RNA was isolated from mouse livers using the Qiagen miRNeasy kit. microRNA levels were detected and normalized to snoRNA412 levels using Taqman miRNA RT-PCR following the manufacturer's instructions (Applied Biosystems, snoRNA412 #1243, miR-122a #2245, miR-378* #567). Real-Time PCR reactions were carried out in a Corbett Research Rotor-Gene instrument.

### Western blot analysis

Nuclear extracts were prepared from livers of WT and ERRα-null mice collected during a 12∶12 h LD schedule. Briefly, the livers were homogenized in cell lysis buffer (HEPES 5 mM, KCl 85 mM, NP40 0.5%) containing protease and phosphatase inhibitor cocktails (Roche) and the nuclei collected were prepared in lysis buffer (sodium phosphate 20 mM, NaCl 150 mM, NP40 1%, EDTA 5 mM, PMSF 1 mM) containing protease and phosphatase inhibitor cocktails (Roche). Equal amounts of protein were pooled from 4 WT and 4 ERRα-null liver nuclear extracts at the indicated time points and a total of 20 µg protein extract at each time point were used for immunoblot analysis. Immunoblot detection was done using a custom made anti-ERRα (1∶10,000) antibody [29], anti-BMAL1 (Santa Cruz, sc-8550X, 1∶1,000) and anti-CLOCK (Santa Cruz, sc-6927X, 1∶1,000) antibodies.

Whole cells lysates from the HepG2 knock-down samples were prepared in lysis buffer (sodium phosphate 20 mM, NaCl 150 mM, NP40 1%, EDTA 5 mM, PMSF 1 mM) containing protease and phosphatase inhibitor cocktails (Roche). 50 ug of total protein lysate was used for immunoblot analysis. Immunoblot detection was done using anti-PROX1 (Proteintech Group, 51043-1-AP, 1∶400) antibody and anti-RPLP (Proteintech Group, 11290-2-AP, 1∶2,000) antibody was used as a loading control.

### Locomotor activity in running wheels

Wheel-running activity data were collected from male adult age-matched WT (n = 9) and ERRα-null (n = 10) mice. Animals were put in running wheel cages (Actimetrics, Wilmette, IL, USA) set in a light-proof ventilated cabinet. Activity under a 12 hr light (∼200 lux), 12 hr dark cycle (LD) was recorded over 5 days following entrainment of the animals to this schedule. Animals were then kept in DD conditions for 20 days and the recordings of day 3 to day 20 in DD were used for defining circadian parameters in free running conditions. All data were analyzed using the Clocklab program (Actimetrics, Wilmette, IL, USA). Chi^2^ periodogram analysis was used for measurement of the free-running period.

### Metabolic measurements

Serum metabolic measurements of insulin, bile acid, total cholesterol, NEFA and triglycerides were conducted on male WT and ERRα knock-out mice (n = 4) under basal (fed *ad libitum*) and fasted conditions. For fasting experiments, mice were housed in cages with woodchip bedding instead of corncob bedding and food was removed from cages 24 hrs prior to the start of blood collection every 4 hrs during a 24 hr period. Blood was isolated by cardiac puncture from mice anesthetized with isofluorene gas. Blood was allowed to clot for 45 min in serum separation tubes (Sarstedt, 41.1378.005) with subsequent centrifugation at 5000 rpm for 30 min. Serum was isolated and stored at −80°C until further processing. Total cholesterol, triglyceride, bile acid and NEFA levels were detected by enzymatic colorimetric rate assays performed at IDEXX Laboratories (Markham, Ontario). Specifically, cholesterol CHOD-PAP (Roche), triglyceride GPO-PAP (Roche), total bile acids assay (Diazyme) and VetSpec NEFA (Catachem Inc.) kits were used and samples were run on a Roche Hitachi H917 chemistry analyzer. Radioimmunoassay detection of insulin with rat insulin RIA kits (Millipore) was performed at the Animal Health Diagnostic Center at Cornell University.

Blood glucose levels were measured using a OneTouch Ultra2 glucose meter (LifeScan) on male WT and ERRα knock-out mice. For glucose tolerance tests, mice were housed in cages with woodchip bedding and fasted for 6 hrs prior to intraperitoneal injection of a 20% glucose solution in 0.9% NaCl at 2 mg/g of body weight. Glucose levels were measured prior to glucose administration and at the indicated time points post-injection.

## Supporting Information

Figure S1 Circadian expression of genes involved in transcriptional regulation (A), glycolysis/gluconeogesesis (B) and the TCA cycle and Oxphos (C). Male WT and ERRα-null mice (n = 4) kept in LD conditions were sacrificed at 4 hr intervals over a 24 hr period from ZT 4 to ZT 24. qRT-PCR analysis was performed on RNA isolated from mouse livers and relative expression data normalized to *Arbp* levels are shown as a function of ZT. Data shown are relative to WT expression levels at ZT 4 arbitrarily set to 1. ZT 0 values are a duplicate of ZT 24 shown for clarity. Error bars represent ± SEM. Student's t test was used to compare WT and ERRα KO liver expression data at the indicated time points, *P<0.05.
(PDF)

Figure S2 Binding profiles of BMAL1 on extended promoters of a subset of clock gene targets obtained from ChIP-on-chip. Putative BMAL1/CLOCK binding sequences (E-boxes) are shown.
(PDF)

Figure S3 Mouse liver ERRα and BMAL1 standard ChIP assays on shared ChIP-on-chip target genes involved in AMPK signaling, glycolysis/gluconeogenesis and insulin receptor signaling.
(PDF)

Figure S4 HepG2 cells treated with either control siRNA (siCtrl) or siRNA against Prox1 were grown in DMEM containing 10% fetal bovine serum (FBS) for 48 hrs then starved in DMEM containing 0.5% fetal bovine serum for 24 hrs. On the day of serum shock, 50% horse serum was added (T = 0) for 2 hrs, and then the medium was changed back to starvation medium. Cells were harvested every 4 hrs during a 24 hr period for total RNA extraction. qRT-PCR analysis of molecular clock genes (A) and involved in glucose homeostasis (B) was performed and relative expression data normalized to *Hprt1* levels are shown as a function of time. Data shown are relative to siCtrl expression levels at T = 0 arbitrarily set to 1. Error bars represent ± SEM. Student's t test was used to compare siCtrl and siProx1 expression data at the indicated time points, *P<0.05.
(PDF)

Figure S5 Venn Diagram illustrating the overlap in ERRα, PROX1, and BMAL1 ChIP-on-chip target genes known to display circadian rhythmic expression profiles under basal conditions in mouse liver.
(PDF)

Table S1 Mouse liver BMAL1 enriched ChIP-on-chip target genes.
(XLS)

Table S2 Comparison of mouse liver ERRα, PROX1 and BMAL1 enriched ChIP-on-chip target genes.
(XLS)

Table S3 Overlap between mouse liver ERRα, PROX1 and BMAL1 enriched ChIP-on-chip target genes with genes previously known to display hepatic circadian rhythms.
(XLS)

Table S4 Mouse primers used for ERRα/PROX1 ChIP quantitative PCR analysis.
(PDF)

Table S5 Mouse primers used for BMAL1/CLOCK ChIP quantitative PCR analysis.
(PDF)

Table S6 Mouse primers used for qRT-PCR.
(PDF)

Table S7 Human primers used for qRT-PCR.
(PDF)

Dataset S1 Mouse liver BMAL1 ChIP-on-chip enriched binding events in BED format which can be loaded into the UCSC mm8 genome browser.
(TXT)
