# Supplementary figures and images for: Genomic Convergence among ERRα, PROX1, and BMAL1 in the Control of Metabolic Clock Outputs
Source: PLoS Genet. 2011 Jun 23;7(6):e1002143. doi: 10.1371/journal.pgen.1002143 (PMC3121748; doi:10.1371/journal.pgen.1002143)

Figure S1

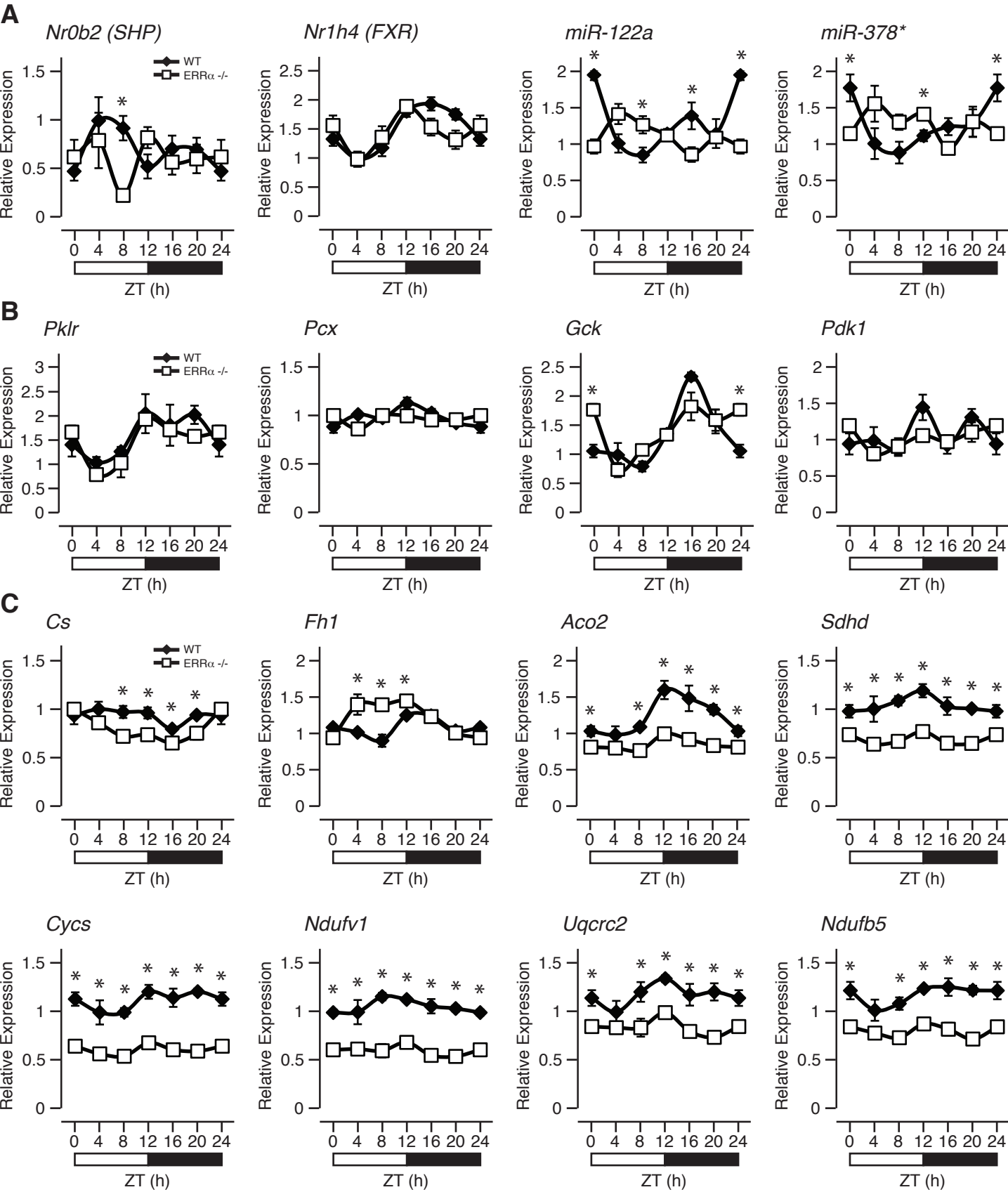

Supplement: Figure S1 — Circadian expression of genes involved in transcriptional regulation (A), glycolysis/gluconeogesesis (B) and the TCA cycle and Oxphos (C). Male WT and ERRα-null mice (n = 4) kept in LD conditions were sacrificed at 4 hr intervals over a 24 hr period from ZT 4 to ZT 24. qRT-PCR analysis was performed on RNA isolated from mouse livers and relative expression data normalized to Arbp levels are shown as a function of ZT. Data shown are relative to WT expression levels at ZT 4 arbitrarily set to 1. ZT 0 values are a duplicate of ZT 24 shown for clarity. Error bars represent ± SEM. Student's t test was used to compare WT and ERRα KO liver expression data at the indicated time points, *P<0.05. (PDF) [file pgen.1002143.s001.pdf]

**Figure S2**

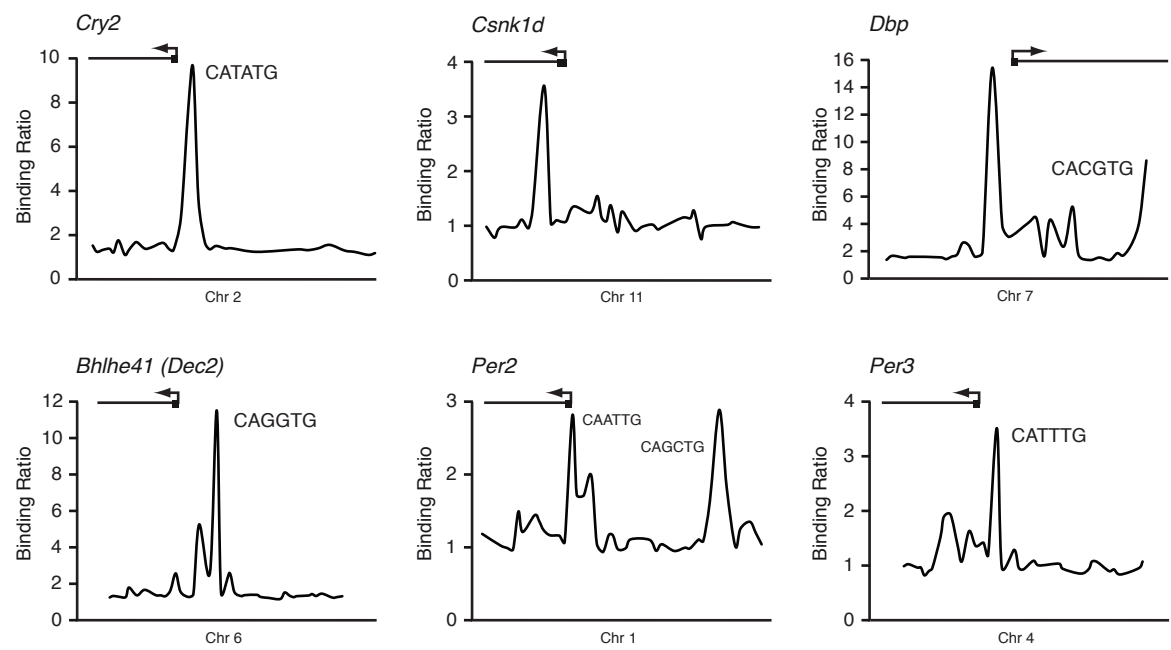

Supplement: Figure S2 — Binding profiles of BMAL1 on extended promoters of a subset of clock gene targets obtained from ChIP-on-chip. Putative BMAL1/CLOCK binding sequences (E-boxes) are shown. (PDF) [file pgen.1002143.s002.pdf]

Figure S3

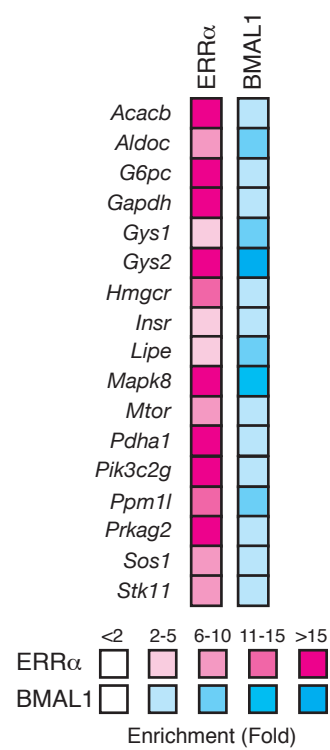

Supplement: Figure S3 — Mouse liver ERRα and BMAL1 standard ChIP assays on shared ChIP-on-chip target genes involved in AMPK signaling, glycolysis/gluconeogenesis and insulin receptor signaling. (PDF) [file pgen.1002143.s003.pdf]

**Figure S4**

**A**

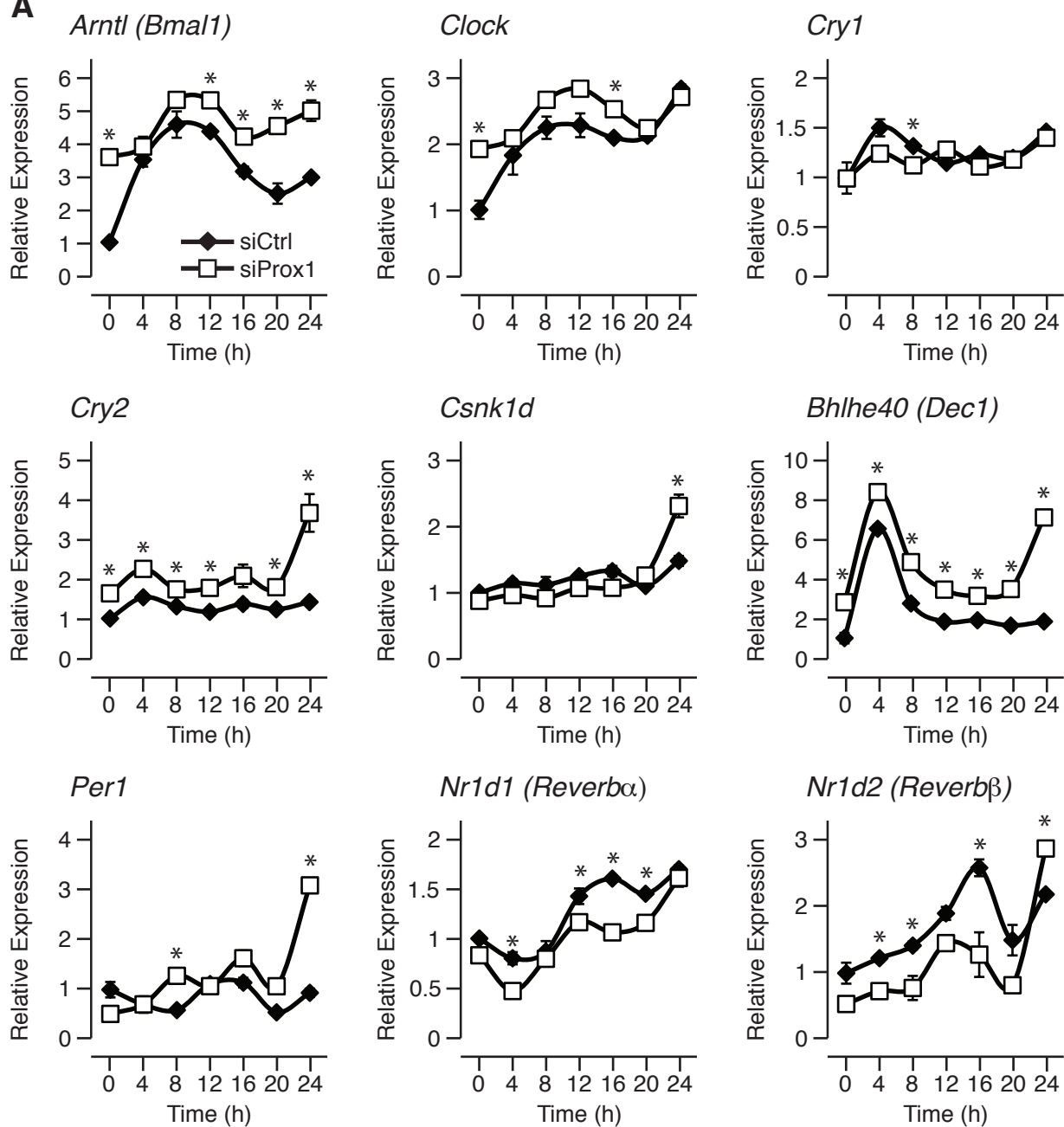

**B**

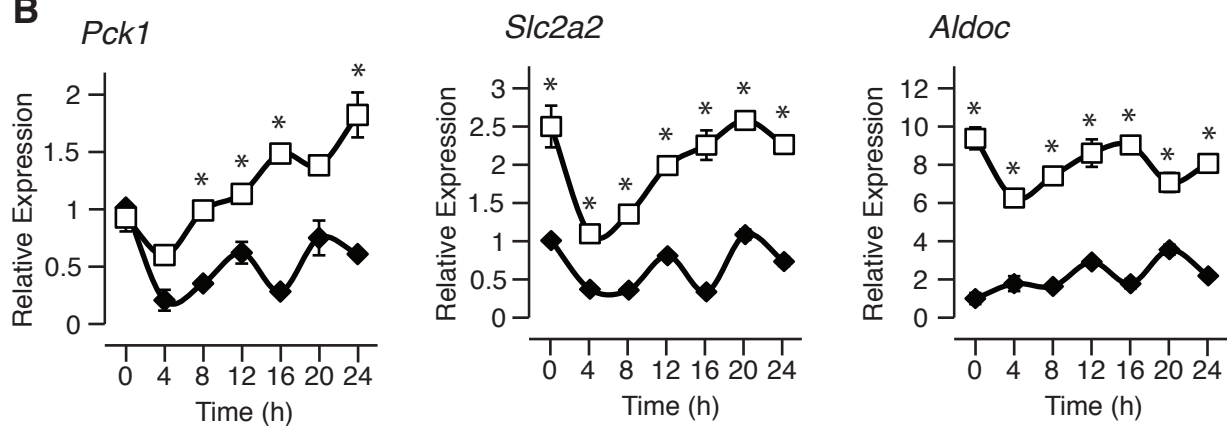

Supplement: Figure S4 — HepG2 cells treated with either control siRNA (siCtrl) or siRNA against Prox1 were grown in DMEM containing 10% fetal bovine serum (FBS) for 48 hrs then starved in DMEM containing 0.5% fetal bovine serum for 24 hrs. On the day of serum shock, 50% horse serum was added (T = 0) for 2 hrs, and then the medium was changed back to starvation medium. Cells were harvested every 4 hrs during a 24 hr period for total RNA extraction. qRT-PCR analysis of molecular clock genes (A) and involved in glucose homeostasis (B) was performed and relative expression data normalized to Hprt1 levels are shown as a function of time. Data shown are relative to siCtrl expression levels at T = 0 arbitrarily set to 1. Error bars represent ± SEM. Student's t test was used to compare siCtrl and siProx1 expression data at the indicated time points, *P<0.05. (PDF) [file pgen.1002143.s004.pdf]

Figure S5

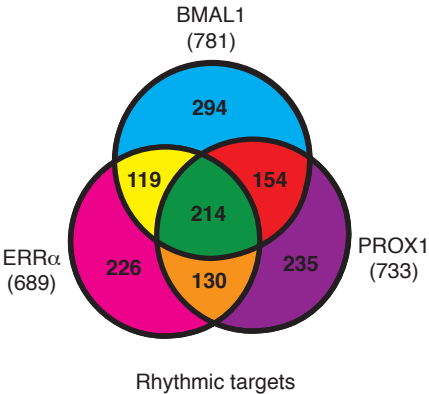

Supplement: Figure S5 — Venn Diagram illustrating the overlap in ERRα, PROX1, and BMAL1 ChIP-on-chip target genes known to display circadian rhythmic expression profiles under basal conditions in mouse liver. (PDF) [file pgen.1002143.s005.pdf]
