# Supplementary material for: Genomic Convergence among ERRα, PROX1, and BMAL1 in the Control of Metabolic Clock Outputs
Source: PLoS Genet. 2011 Jun 23;7(6):e1002143. doi: 10.1371/journal.pgen.1002143 (PMC3121748; doi:10.1371/journal.pgen.1002143)
Supplement: Table S4 — Mouse primers used for ERRα/PROX1 ChIP quantitative PCR analysis. (PDF) [file pgen.1002143.s009.pdf]

Supplemental table 4

Mouse primers used for ERRα/PROX1 ChIP quantitative PCR analysis.

GENE	PRIMER
Acacb (ERRα)	forward 5'-GACCTGTCACTTGACATTGAGTCACC-3'<br>reverse 5'-GGCACGGGTCTAGTTCTGGGTACG-3'
Aldoc (ERRα)	forward 5'-CCTTGATGGTATAGTTTGTCCTTGGG-3'<br>reverse 5'-GCACGAGTCAGGGCTGGAGTTC-3'
Bmal1	forward 5'-CCTCGGGGCGTGTGCTTCTGTG-3'<br>reverse 5'-GCCAATCAGAGAGAGCGAACG-3'
Clock (ERRα)	forward 5'-CAGCAATGTGAACTAGGAGGAGAATC-3'<br>reverse 5'-GGAGAATGGGTGGGTTCAAAGG-3'
Clock (Prox1)	forward 5'-CTCGTGAATGTGTTTGTTTTATTGCTTA-3'<br>reverse 5'-GAATAAAGCCAAAACATTTGACCCAGGG-3'
Cry1	forward 5'-GGACGTTGACAGACTTCTCCCTTGTTC-3'<br>reverse 5'-CAAACGCTAATGGTCTGGACTCATATCC-3'
Cry2	forward 5'-CACAAAGGAGTAGGCAGTCATAGATAG-3'<br>reverse 5'-CAAATGACCATCTCTGCTGTGG-3'
Csnk1d (ERRα)	forward 5'-CTCGATGGTCAGAAGGAGGCAGTG-3'<br>reverse 5'-GCGATGGCTTTGGCACACACATTC-3'
Csnk1d (Prox1)	forward 5'-GAGGGTGTAGAGGGTATGTCTCAGACTC-3'<br>reverse 5'-GGTCAGAATGGACAGCCAAGTCTTG-3'
Dec1 (Bhlhe40)	forward 5'-CCCTGTTAGACTTTCTGCTGAAGGC-3'<br>reverse 5'-GTTCACTGAGCCCTTTACCCAGTCG-3'
ERRα control	forward 5'-TTGGCATTGATATTGGGGGTGGGAGCAACT-3'<br>reverse 5'-GACTTCTTACTTTGACGCTTTCCTCCATCG-3'
G6pc (ERRα)	forward 5'-GCCTCCCCCATAGATTGGTTGGTCGG-3'<br>reverse 5'-CCTCCCCCTCCTGAGTATTGGCATTAC-3'
Gapdh (ERRα)	forward 5'-CTCATTCATCAGCAAGCTCAAAGG-3'<br>reverse 5'-CCACATGTTTTCTCAGTCTTTCCC-3'
Gys1 (ERRα)	forward 5'-GTGGGTTCCTGACGTCTCTTGCTC-3'

	reverse 5'-CGTGTGGTTGCTACAAAATGTGCTC-3'
Gys2 (ERRα)	forward 5'-GACTATTCACAGCAGGGCAGGTAGC-3'<br>reverse 5'-GAGAGTGTAGGAGTTTGGTCTAAAGGC-3'
Hmgcr (ERRα)	forward 5'-GGAATGTGACCTCTGGAACCTGGC-3'<br>reverse 5'-CCTCTTTTGTGATGGAGAATCAGAAGC-3'
Insr (ERRα)	forward 5'-GCTACGGGAGTCTTCAACCTTC-3'<br>reverse 5'-CCCATTGCTCCATACAACTTAG-3'
Lipe (ERRα)	forward 5'-CCCTAGCCAACTATGACCCAGTCC-3'<br>reverse 5'-CTTCTCAGGGCAAAGGGCTGGAG-3'
Mapk8 (ERRα)	forward 5'-GTGTTTGAAGGTCAGGCACGC-3'<br>reverse 5'-GAGCTAGAGAACATCTACTCCTTGGTGG-3'
Mtor (ERRα)	forward 5'-CCCTGTAGTGTAGCGTTTGAAAGCC-3'<br>reverse 5'-CCAGCACTGAGGGAGAGGCAAG-3'
Pdha1 (ERRα)	forward 5'-GTGTGGCTAGAGGTCAAAATGCTAAAAG-3'<br>reverse 5'-CACACAGAGGCTTAGAGTTCAAAGTAGCG-3'
Per1	forward 5'-CTGAGGGAGGGTAAAAGTAGGTC-3'<br>reverse 5'-GGACATGCACACAGCCTTGG-3'
Per2 set1	forward 5'-GCTGACAGAGAACAACCTAAAGACTC-3'<br>reverse 5'-CCTCCTTTCCATTCCTGGTTCC-3'
Per2 set2	forward 5'-CAGGAATCATGCCACCGATG-3'<br>reverse 5'-GGAGCTTGGACTTTGCACAACC-3'
Per2 set3	forward 5'-GACAGACCCTGGTGGTGGCTG-3'<br>reverse 5'-GCCTATTCCATGCAACACCTG-3'
Pik3c2g (ERRα)	forward 5'-CGCTCTCCAACAGTTAAAAGAAAGTTTGC-3'<br>reverse 5'-GCGTTCCCTTTTCCAAATTTCATCTAAG-3'
Ppm1l (ERRα)	forward 5'-GCTGAGAGACACGAGAGACACTGGATATTC-3'<br>reverse 5'-GCCTGGTGACAAAGTTCACTGCATATAC-3'
Prkag2 (ERRα)	forward 5'-CTGATACCAGAGTATCGGGTTGGAAGTAC-3'<br>reverse 5'-CCTGTTTAACTGGGTAACCTTTGTTTCC-3'
Prox1 control	forward 5'-CCAAGCACAAATATCTAATCACCCTTTC-3'<br>reverse 5'-CTTCTTGATAGGTTTATGGGTTGGGC-3'

Reverbα (ERRα)	forward 5'-GGTTTCACGGCTGGGTTGGAGG-3'<br>reverse 5'-GGAGAGCTAGGGTCTTTTGAGCATTGCC-3'
Reverbα (Prox1)	forward 5'-GGCAAGGGCATGTGAATTCCTG-3'<br>reverse 5'-GGAAATCTCTCCTCCCAGCTTG-3'
Reverbβ	forward 5'-CTCATCTGTGAGACAAGCAAGGAAAG-3'<br>reverse 5'-GGCCTGTCATCTGATATGTTACAGTTTC-3'
Sos1 (ERRα)	forward 5'-CGGACTCCTAAAACGAGAGGTTGACC-3'<br>reverse 5'-GGTTGGAGGAGCCTCGGTTTTC-3'
Stk11 (ERRα)	forward 5'-GGGTAGAAACAGAATGTTGTCTATTGCC-3'<br>reverse 5'-GAAGTCTCCTCCTCCACCTCCAC-3'
