# Supplementary material for: Genomic Convergence among ERRα, PROX1, and BMAL1 in the Control of Metabolic Clock Outputs
Source: PLoS Genet. 2011 Jun 23;7(6):e1002143. doi: 10.1371/journal.pgen.1002143 (PMC3121748; doi:10.1371/journal.pgen.1002143)
Supplement: Table S5 — Mouse primers used for BMAL1/CLOCK ChIP quantitative PCR analysis. (PDF) [file pgen.1002143.s010.pdf]

Supplemental table 5

Mouse primers used for BMAL1/CLOCK ChIP quantitative PCR analysis.

GENE	PRIMER
Acacb	forward 5'-GACCTGTCACTTGACATTGAGTCACC-3'<br>reverse 5'-GGCACGGGTCTAGTTCTGGGTACG-3'
Aldoc	forward 5'-CCCAGGGAGTCACGTAGCTCTG-3'<br>reverse 5'-GCCACAGCACAGCTCACTTTTCC-3'
Bmal1	forward 5'-GATTGGTGAAGGGAAAGTAGCAGG-3'<br>reverse 5'-GAGAGCGAACGGAGTTGTCTTGG-3'
Clock	forward 5'-GCTAGATTCCTTTGGAAAGTTCAGTTAAAG-3'<br>reverse 5'-CCAGCCTCAGTACCCTTACCTCCTG-3'
Cry2	forward 5'-GTGGCTGACAGACCTGTTACCG-3'<br>reverse 5'-GAAAATAGGACAGTTTGTGTAAGGCAATC-3'
Dec1 (Bhlhe40) site1	forward 5'-CTTCCCAAAGGTCCTTGTAAATTC-3'<br>reverse 5'-CATTTATGCAGCACAAGTTCACTGAG-3'
Dec1 (Bhlhe40) site2	forward 5'-GCACTTCGCAGCCGCCAGAG-3'<br>reverse 5'-GTTCTACCCTGTGACTCCAAGCACGTC-3'
Esrra	forward 5'-CCTCGAACCCAAGCTCAACCTCATC-3'<br>reverse 5'-GGACAAAACTCAAGGTCACTGCGGTG-3'
G6pc	forward 5'-GCCTCTTCACGAGCAACCCTTATC-3'<br>reverse 5'-GAGCAGGGCTGTCTGTGTCAGG -3'
Gapdh	forward 5'-CTCATTCATCAGCAAGCTCAAAGG-3'<br>reverse 5'-CCACATGTTTTCTCAGTCTTTCCC-3'
Gys1	forward 5'-GTGGGTTCCTGACGTCTCTTGCTC-3'<br>reverse 5'-CGTGTGGTTGCTACAAAATGTGCTC-3'
Gys2	forward 5'-GCGGAAGCCAGGACAGAGTG-3'<br>reverse 5'-CTCTCACACATGCACCCACGG-3'
Hif1a	forward 5'-GAGAGGTTTGGCAAGACGGATTTG-3'<br>reverse 5'-GACCGACACCACTCAGTCTCCAGTC-3'
Hmgcr	forward 5'-GGAATGTGACCTCTGGAACCTGGC-3'

	reverse 5’-CCTCTTTTGTGATGGAGAATCAGAAGC-3’
Hnf4a	forward 5’-CCCTAGTATCTCTTGCCAAGTACCATG-3’<br>reverse 5’-CCTGAGTAAACCCCTCTGAATCACC-3’
Insr	forward 5’-CTTTACAGCCTATGCTACCAATGTCTTAC-3’<br>reverse 5’-CTCACTGGGGAGGGAACTTG-3’
Irf8	forward 5’-GGACGTGCAAAAGTGATTTCTCGG-3’<br>reverse 5’-CCCAATCAGCGCAGCCTGCC-3’
Lipe	forward 5’-GCTGCCCTGGGAGGATTAAGCC-3’<br>reverse 5’-CAAAAGCCTGAGAGGTTTCTCCGAG-3’
Mapk8	forward 5’-GTGTTTGAAGGTCAGGCACGC-3’<br>reverse 5’-GAGCTAGAGAACATCTACTCCTTGGTGG-3’
Mtor	forward 5’-CGGTCACAGCGTGTGTGGAGG-3’<br>reverse 5’-GACTATTCCGAAGAGCCAATCAGTCGTG-3’
Nr6a1	forward 5’-CGGCTATGTCAGAGTTTTCTCTCTATTG-3’<br>reverse 5’-GTGGATACGCCTCCTTGGAAAG-3’
Pdha1	forward 5’-GCTTCTTGATGGTTCCTGGTGC-3’<br>reverse 5’-GTCATTGCTCTTGGCTATGAGGC-3’
Per1 site1	forward 5’-GGTCAGATGTCCAATCAACGAG-3’<br>reverse 5’-GGCATTTCAGAACTCAGTCATTTTCC-3’
Per1 site2	forward 5’-GGTAGTTTCCCTCCCTCACTTCCC-3’<br>reverse 5’-CCTGGCATCTGATTGGCTACTGGC-3’
Pik3c2g	forward 5’-CTCATTGCCTGCCCTGCTAAGC-3’<br>reverse 5’-CATAAGTGACTGGTACGACTACTCTCTCAC-3’
Ppargc1a	forward 5’-GCAATTCAATTCGGGTCCATCTCACC-3’<br>reverse 5’-GGACTTCAGCGTGTTTGCATTCAGTAC-3’
Ppargc1b	forward 5’-CCGACCCTGGCTGGATTCTACC-3’<br>reverse 5’-GCTGTAGCATTGGGGGACACAG-3’
Ppm1l	forward 5’-CCGTACCATTGTGTAGCGTGCC-3’<br>reverse 5’-GGGACAATGTGATAGTGACGTGAGGTC-3’
Prkag2	forward 5’-CATGGTTGTCACTCTGGTCCCCAC-3’<br>reverse 5’-CACTTAAGGTTCAGTGCTTAGCAGACAAAC-3’

Prox1	forward 5'-CTTACATAACCCAGCCACTGCAC-3'<br>reverse 5'-GCAGATGAACAAGCAATCTCAGGG-3'
Reverbα site1	forward 5'-CCGTGGAAGATCAAAGCTAAAAGGC-3'<br>reverse 5'-CTCCTCCCAGCTTGTCTTTCTCCC-3'
Reverbα site2	forward 5'-GGAAGTAGGCTAGAACTGTAGAAAAGGG-3'<br>reverse 5'-GAGTTTAGGGAGACTCAGCATTTTGAAGC-3'
Sos1	forward 5'-CTGCTTTTCTGTTCCAGATCCACTG-3'<br>reverse 5'-CAGATGTATTTGGACCGATAAGTAGAGGTG-3'
Stk11	forward 5'-GACAACCAATGAGAATTTAGATCACGC-3'<br>reverse 5'-CAACGAGGATTGGGCTATTTGGATCTAG-3'
