# Supplementary material for: Genomic Convergence among ERRα, PROX1, and BMAL1 in the Control of Metabolic Clock Outputs
Source: PLoS Genet. 2011 Jun 23;7(6):e1002143. doi: 10.1371/journal.pgen.1002143 (PMC3121748; doi:10.1371/journal.pgen.1002143)
Supplement: Table S6 — Mouse primers used for qRT-PCR. (PDF) [file pgen.1002143.s011.pdf]

Supplemental table 6

Mouse primers used for qRT-PCR.

GENE	PRIMER
Abcb11	forward 5'-GAGTACACACCAGGGACACTGATCCAG-3'<br>reverse 5'-GCTTGTAACCATCTCCTGACATGCAGTC-3'
Acacb	forward 5'-GGGCTCCCTGGATGACAACTC-3'<br>reverse 5'-GACGGTGAAATCTCTGTGCAGG-3'
Acadm	forward 5'-GGGGAGGATGACGGAGCAGC-3'<br>reverse 5'-CGGGTACTTTAGGATCTGGG-3'
Aco2	forward 5'-CTTTCGACAAGTGGGACGGCAAAGAC-3'<br>reverse 5'-GACAGCATTGCGTACAGAGTTGGC-3'
Arbp	forward 5'-GCAGCAGATCCGCATGTCGCTCCG-3'<br>reverse 5'-GAGCTGGCACAGTGACCTCACACGG-3'
Bmal1	forward 5'-CTCACCGTGCTAAGGATGGCTGTTC-3'<br>reverse 5'-GACTCGGAGACAAAGAGGATCTTCCC-3'
Clock	forward 5'-GGGCACCACCAATAATAGGCTATTTGC-3'<br>reverse 5'-GAGTCTGAAGCCATATCCACTGCTG-3'
Cry1	forward 5'-GACACCATCCGCTGCGTCTATATC-3'<br>reverse 5'-CTTGAAAAGCCTGGGAAATACATCAG-3'
Cs	forward 5'-CCCTGAGTGCCAGAAAATGCTG-3'<br>reverse 5'-CCACATGAGAAGGCAGAGCTGC-3'
Cycs	forward 5'-CCAAATCTCCACGGTCTGTTCG-3'<br>reverse 5'-GTATCCTCTCCCCAGGTGATGC-3'
Cyp7a1	forward 5'-CACAAACTCCCTGTCATACCACAAAGTC-3'<br>reverse 5'-GTGAACACAGAGCATCTCCCTGGAGG-3'
Cyp8b1	forward 5'-CCAGTACTTCACCTTTGTCATGGAC-3'<br>reverse 5'-GTACCCAAACACCTTGAGCACCAGTTC-3'
Cyp27a1	forward 5'-GAAGGACCACCGAGACCACAAGG-3'<br>reverse 5'-CGTTTAAGGCATCCGTGTAGAGCG-3'
Dbp	forward 5'-GCAAGGAAAGTCCAGGTGCCTGAG-3'<br>reverse 5'-CACCACCTCCTGCCGCAACAG-3'

Dhcr7	forward 5’-GACTTCTGCCACAGATTTCTGCCAGG-3’<br>reverse 5’-GGGTAATGAGCCAGGCTTGCAGC-3’
Esrra	forward 5’-CTCAGCTCTCTACCCAAACGC-3’<br>reverse 5’-CCGCTTGGTGATCTCACACTC-3’
Esrrg	forward 5’-CTCCAGCACCATCGTAGAGGATC-3’<br>reverse 5’-GATCTCACATTCATTCGTGGCTG-3’
Fh1	forward 5’-GCAGTGGAAGTTCACAAGGTCCTG-3’<br>reverse 5’-CTGGACTTGCTGAACGTAACCAC-3’
G6pc	forward 5’-GCTCACTTTCCCCACCAGGTCG-3’<br>reverse 5’-CACCCCTAGCCCTTTTAGTAGCAGGTAG-3’
Gck	forward 5’-GCCACAATGATCTCCTGCTACTATGAAG-3’<br>reverse 5’-CACCAGCTCCACATTCTGCATC-3’
Glut2	forward 5’-GCCATCAACATGATCTTCACGGCTG-3’<br>reverse 5’-CAGTCATGCTCACGTAACTCATCCAGG-3’
Gys1	forward 5’-CGGTTGTCGGACTTGCTAGATTGG-3’<br>reverse 5’-CATAGGTGAAGTGGTCTGGAAAGGC-3’
Gys2	forward 5’-CCTTGGGGTGTTTCCATCGTAC-3’<br>reverse 5’-CGGAGAGGTTTGTAGTCACACTGG-3’
Hmgcr	forward 5’-GCTCTTGTGGAATGCCTTGTGATTG-3’<br>reverse 5’-CTCTAGGACCAGCGACACACAGG-3’
HSD3B7	forward 5’-GACCATCCACAAAGTCAACGTGC-3’<br>reverse 5’-GCCTCATATGGGGTATCTTCATTGCC-3’
Insr	forward 5’-GCTGATGGCATGGCATACTTGAAC-3’<br>reverse 5’-CCTCACAGGAAGCAGTCCCTTG-3’
Lipe	forward 5’-GACCTCCGACTGGGTGCCTC-3’<br>reverse 5’-GCTGCCTCAGACACACTCCTGC-3’
Lipc	forward 5’-CTGCCACTTCCTGGAACTCTACAAACAC-3’<br>reverse 5’-GCTGCCCATGTCGCTGCACTG-3’
Mlycd	forward 5’-CCGACTGAAACCGAGGAGAGG-3’<br>reverse 5’-CAGCAGCCACTTGGTGAATCC-3’

Ndufb5	forward 5’-CGAGCTTGCAGAAATCCCAGAAGGC-3’<br>reverse 5’-GTCCATCACCTCGGGCACGCATCAG-3’
Ndufv1	forward 5’-GCTAATGGACTTCGATGCACTGG-3’<br>reverse 5’-CACAGCTCTCATGCTTGTAGAACTCAATG-3’
Nr0b2 (Shp)	forward 5’-CCAGGGTGCCCAGCCATCAG-3’<br>reverse 5’-CCCAGTGAGCCTCCTGTTGCAG-3’
Nr1h4 (Fxr)	forward 5’- GGAGTGGTAGCCAAGATGAATCTGATTG-3’<br>reverse 5’- GGAAACGGGACATTGTTGTATGGG-3’
Pck1	forward 5’-CCAGGCAGTGAGGAAGTTCG-3’<br>reverse 5’-GAGGGTCAGTGAGAGCCAGC-3’
Pcx	forward 5’-GGTGTTCTTTGAACTCAATGGGCAGC-3’<br>reverse 5’-CCTTGGTAACATGAACCTTTCGGATAGTG-3’
Pdha1	forward 5’-GCCGAGTGCTGGTTGCTTCCC-3’<br>reverse 5’-GTCTGCATCATCCTGTAGTACTTGAGCC-3’
Pdk1	forward 5’-GTGTTGAAACGTCCCGTGCTG-3’<br>reverse 5’-GCTTCCAGGCGGCTTTATTGT-3’
Pdk4	forward 5’-GATCCTAACCACCGCCAGCC-3’<br>reverse 5’-GCAAAGGACGTTCTTTCACAG-3’
Pik3c2g	forward 5’-CCCTGAATGAAGAGCTTTCCAAGGAG-3’<br>reverse 5’-GGGTTCAGAGGAAGATGGCAAGTC-3’
Pik3r1	forward 5’-CCCACTACTGTAGCCAACAACAGC-3’<br>reverse 5’-GAGTGTAATCGCCGTGCATTTTAG-3’
Pklr	forward 5’-GAGTCTTCCCCTTGCTCTACCGTGAG-3’<br>reverse 5’-GTATAGCCAGAGCCAGGTCGCCAG-3’
Ppargc1a	forward 5’-CAGTCCTTCCTCCATGCCTG-3’<br>reverse 5’-GGGTTTGTTCTGATCCTGTGG-3’
Ppargc1b	forward 5’-CAGCCAGTACAGCCCCGATG-3’<br>reverse 5’-GGTGTGTCGCCTTCATCCAG-3’
Prkag2	forward 5’-CAGAAAGTGGCGTTTATATGCGATTC-3’<br>reverse 5’-GGCTACCAAGGCAAAGAAGGC-3’
Prox1	forward 5’-CAGCACACCCACCCAGCACCG-3’

	reverse 5’-CACGTCCGAGAAGTAGGTCTTCAG-3’
Reverbα	forward 5’-CTCACTCGTCTCCCTCAGCCATTG-3’<br>reverse 5’-GGAGCCACTAGAGCCAATGTAGGTG-3’
Sdhd	forward 5’-GGTACTTGAATCCCTGCTCTGTGGTG-3’<br>reverse 5’-GTCCCCATGAACGTAGTCGGTAACC-3’
Slc10a1	forward 5’-GCCACACTATGTACCCTACGTCCTC-3’<br>reverse 5’-GAATGTAGCCCATCAGGAAGCCAGTG-3’
Stk11 (Lkb1)	forward 5’-GCAAGGTGAAGGAGGTGCTG-3’<br>reverse 5’-GCTGGATCACATTCCGATGC-3’
Uqcrc2	forward 5’-GCCACCTTCTACCGTCCTTCAACAGATTG-3’<br>reverse 5’-CGTATGTCCCAAGTTTCCACTCGCTGC-3’
