# Supplementary material for: Genomic Convergence among ERRα, PROX1, and BMAL1 in the Control of Metabolic Clock Outputs
Source: PLoS Genet. 2011 Jun 23;7(6):e1002143. doi: 10.1371/journal.pgen.1002143 (PMC3121748; doi:10.1371/journal.pgen.1002143)
Supplement: Table S7 — Human primers used for qRT-PCR. (PDF) [file pgen.1002143.s012.pdf]

Supplemental table 7

Human primers used for qRT-PCR.

GENE	PRIMER
Bmal1	forward 5'-GCCATCTCGATTATGTTCTGGAGCAC-3'<br>reverse 5'-CATTGTCTGGTTCGTTGTCTTCATCC-3'
Clock	forward 5'-GCAGTAACTACATTCACTCAGGACAGGCAG-3'<br>reverse 5'-GTAGGATATGCAGTCACCACCTGGC-3'
Cry1	forward 5'-GATTCCAGACTCTCATCAGCAAAATG-3'<br>reverse 5'-CCAAACGAGTAAGTGCTTCAGTTTCTCC-3'
Cry2	forward 5'-CAGAGTCAATTCAGAAGGCAGCCAAG-3'<br>reverse 5'-CACAGGAAGGGACAGATGCCAGTAG-3'
Csnk1d	forward 5'-GCTGAAGGCTGCCACCAAGAGACAG-3'<br>reverse 5'-CGCAGGTACGAGTAGTCAGGCTTGTC-3'
Dec1	forward 5'-GATCAGCAGCAGCAGAAAATCATTG-3'<br>reverse 5'-CGAAGACTTCAGGTCCCGAGTGTTC-3'
Hprt1	forward 5'-CTAATCATTATGCTGAGGATTTGG-3'<br>reverse 5'-CTATTCAGTGCTTTGATGTAATCCAGC-3'
Per1	forward 5'-GCCAACCAGGAATACTACCAGCAGTG-3'<br>reverse 5'-GCCTGCTCCGAAATGTAGACGATTC-3'
Reverbα	forward 5'-GAGGATTTCTCCATGAGCTTCACG-3'<br>reverse 5'-GGCTTAGGAACATCACTGTCTGGTCC-3'
Reverbβ	forward 5'-GGCATCTTGAAGAATGATCGAATAGATTG-3'<br>reverse 5'-CCTTACAGCCTTCGCAAGCATGAACTC-3'
